# High-Throughput Sequencing Identifies Novel and Conserved Cucumber (*Cucumis sativus* L.) microRNAs in Response to Cucumber Green Mottle Mosaic Virus Infection

**DOI:** 10.1371/journal.pone.0129002

**Published:** 2015-06-15

**Authors:** H. W. Liu, L. X. Luo, C. Q. Liang, N. Jiang, P. F. Liu, J. Q. Li

**Affiliations:** 1 Department of Plant Pathology/Key Laboratory of Plant Pathology, China Agricultural University, Beijing, People’s Republic of China; 2 Beijing Engineering Research Centre of Seed and Plant Health/Beijing Key Laboratory of Seed Disease Testing and Control, Beijing, People’s Republic of China; 3 Molecular Plant Pathology Laboratory, United States Department-Agricultural Research Service, Beltsville, Maryland, United States of America; East Carolina University, UNITED STATES

## Abstract

Seedlings of *Cucumis sativus* L. (cv. 'Zhongnong 16') were artificially inoculated with Cucumber green mottle mosaic virus (CGMMV) at the three-true-leaf stage. Leaf and flower samples were collected at different time points post-inoculation (10, 30 and 50 d), and processed by high throughput sequencing analysis to identify candidate miRNA sequences. Bioinformatic analysis using screening criteria, and secondary structure prediction, indicated that 8 novel and 23 known miRNAs (including 15 miRNAs described for the first time *in vivo*) were produced by cucumber plants in response to CGMMV infection. Moreover, gene expression profiles (p-value <0.01) validated the expression of 3 of the novel miRNAs and 3 of the putative candidate miRNAs and identified a further 82 conserved miRNAs in CGMMV-infected cucumbers. Gene ontology (GO) analysis revealed that the predicted target genes of these 88 miRNAs, which were screened using the psRNATarget and miRanda algorithms, were involved in three functional categories: 2265 in molecular function, 1362 as cellular components and 276 in biological process. The subsequent Kyoto Encyclopedia of Genes and Genomes (KEGG) pathway analysis revealed that the predicted target genes were frequently involved in metabolic processes (166 pathways) and genetic information processes (40 pathways) and to a lesser degree the biosynthesis of secondary metabolites (12 pathways). These results could provide useful clues to help elucidate host-pathogen interactions in CGMMV and cucumber, as well as for the screening of resistance genes.

## Introduction

MicroRNAs (miRNA) are evolutionarily conserved endogenous noncoding RNAs [1] that were first discovered in the nematode *Caenorhabditis elegans* [2]. Subsequent studies have revealed that miRNAs can be found in a broad range of species including plants, nematodes, fruit flies, mice and humans. As of June 2014 as many as 30424 miRNAs from 206 species have been deposited in the miRBase database (http://www.mirbase.org, Release 21, June 2014). Empirical investigations have recently demonstrated that miRNAs play important regulatory roles in a range of biological and metabolic processes including the development and differentiation of plant organs, as well as in response to external biotic and abiotic stresses [3]. More specifically miRNAs are known to affect processes such as protein degradation, signal transduction, and responses to pathogen invasion as well as the regulation of their own biogenesis [1]. Their mode of action appears to involve post-transcriptional gene regulation by the repression of translation or cleavage of targeted mRNAs.

Like other species of RNAs, miRNAs are initially formed in the nucleus before being translocated to the cytoplasm. The primary miRNAs (pri-miRNA), which have a characteristic stem-loop structure, are transcribed by RNA polymerase Ⅱ within the plant nucleus, then exported out of the nucleus by Hasty (HST) [4]. In the cytoplasm the pre-miRNAs are cleaved into miRNA by the enzyme Dicer to form 22 nt miRNA* duplexes. The resulting miRNA duplexes are integrated into the RNA-induced silencing complex (RISC), which recognizes complementary sites on the target mRNAs resulting in transcript cleavage of the regulated genes [5]. To date, the main approaches to identifying miRNAs in plants have been direct cloning and sequencing by forward genetics, and bioinformatics methods, which have been applied extensively to explore novel and conserved miRNAs in *Arabidopsis thaliana* and *Oryza sativa* [6, 7]. However, the development of high-throughput sequencing methods using a Solexa platform has offers several advantages over conventional Sanger sequencing and can produce more abundant readings as a result of its increased sensitivity to short nucleic acid fragments [8]. As a consequence, it has been suggested that many novel and conserved plant miRNAs could be discovered using the Solexa system [9].

Cucumber (*Cucumis sativus* L.), which originated in India, is an economically important member of the gourd family (*Cucurbitaceae*) and is cultivated worldwide [10]. However, the production of crops belong to the cucurbitaceae has recently become increasingly threatened by the *Cucumber green mottle mosaic virus* (CGMMV), which belong to the *Tobamovirus* genus of the *Virgaviridae* family. Plants infected with CGMMV produce several characteristic symptoms, including mosaic patterning on the leaves, and fruit distortions [11, 12]. The reduced yield and lower market value of affected fruits causes substantial economic losses worldwide [13]. More frequent trade between different geographic regions has led to the rapid spread of CGMMV via contaminated materials, which include propagation stock, seeds, soil and pollen [12, 14–18].

In addition to being a high value crop, cucumber is also an important model system for plants, and has been used as a subject in many areas of plant research including induced and acquired resistance to disease [19], sex-determining mechanisms [20], and phloem biology [21, 22]. The commercial importance of cucumber has also resulted in it being selected as the seventh plant genome to be completely sequenced, following *Arabidopsis thaliana*, *Poplar trichocarpa*, *Grapevine* (*Vitis vinifera*), *Papaya* (*Carica papaya*), and the field crops *Oryza sativa* and *Sorghum bicolor* [23, 24]. Furthermore, genetic and genomic variation maps have also been produced for cucumber [25, 26], all of which are useful resources to study the function of miRNAs in response to pathogen infection. Indeed a recent study using high-throughput sequencing isolated 19 conserved sequences, as well as 7 novel candidate miRNAs from cucumbers in response to infection with *Hop stunt viroid* [27]. Meanwhile a similar study identified 29 miRNAs produced by healthy cucumber plants that target mRNAs associated with a broad range of physiological processes including development and signaling transduction as well as transcriptional regulation [28]. However, to date there has been little investigation of the post-transcriptional role of miRNAs in cucumber. The identification of a greater number of miRNAs is a prerequisite to further our understanding of miRNA function, while miRNAs associated with pathogenic infections are of particular interest as they are thought to have significant roles in regulatory functions [29, 30].

Even though it is extremely likely that miRNAs have important roles in virus resistance, to date there have been no reports regarding the role of miRNAs in cucumber plants infected by viruses. The objective of the current study was therefore to characterize the developmental and organ-specific miRNAs produced by cucumbers in response to CGMMV infection using next generation sequencing and gene expression profiles, and in conjunction with GO and KEGG pathway analysis, to ascribe possible functions to the miRNAs identified.

## Materials and Methods

### Sample collection and total RNA isolation

Cucumber seeds (cv. Zhongnong 16 [31]) were obtained from the Institute of Vegetables and Flowers at the Chinese Academy of Agricultural Sciences (Beijing, China) and confirmed to be CGMMV-negative by RT-PCR. The seeds were germinated and planted in accordance with a previous study [11] and kept under insect-proof netting (Zhiguang wire mesh products, Hebei, China) to ensure the plants remained virus free. The CGMMV inoculum was prepared from infected cucumber leaves obtained from the Chinese Academy of Inspection and Quarantine (CAIQ), Beijing, China.

**Fig 1:**
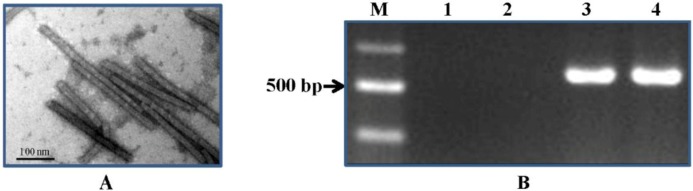
Confirmation of CGMMV-infection in cucumber samples (cv. Zhongnong 16). A, CGMMV particles in infected cucumber leaves observed by SEM. B. Gel electrophoresis of RT-PCR using CGMMV specific primers. M, DNA ladder; 1, Sample from uninfected plants; 2, Blank control; 3, Sample from infected plant; 4, Infected leaves obtained from CAIQ used as a positive control.

Cucumber seedlings were inoculated with CGMMV at the three-true-leaf stage. Leaves were collected at 10, 30 and 50 days post inoculation (dpi). The leaves from the inoculated plants were confirmed to be infected with CGMMV using SEM and RT-PCR before their RNA was extracted and sequenced (Fig 1). At anthesis the flowers (both male and female) were also collected. Non-inoculated cucumber leaves and flowers were used as negative controls. All the samples were immediately snap-frozen in liquid nitrogen and stored at -80°C until required. Total RNA was extracted from the leaf and flower samples (three leaves or flowers, which were collected from different plants, were pooled as one leaf or flower sample) using the EASYspin Kit (Biomed, Beijing, China) according to the protocol of the manufacturer. Genomic DNA was removed from the samples by treatment with RNase-free DNase I (TaKaRa, Dalian, China). The quality of the resulting RNA was assessed using the Agilent 2100 Bioanalyzer system (Agilent Technologies, Inc., USA).

### Construction and sequencing of small RNA library

Small RNA (sRNA) fragments (10–40 nt) were isolated from the total RNA samples (200 μg) using 15% (w/v) denaturing polyacrylamide gel electrophoresis (PAGE), and purified using a Small RNA Gel Extraction Kit (Life technologies, USA). After ligation of the 5' and 3' adaptors, the short RNA fragments were reverse transcribed and amplified by PCR (RT-PCR kit, Invitrogen, USA). The resulting cDNA library was purified using the MinElute PCR Purification Kit (Qiagen, Germany) and its quality assessed using an Agilent 2100 Bioanalyzer (Agilent Technologies, Inc., USA). Finally the entire cDNA library from each sample set was processed using the Cluster Station (Illumina, San Diego, USA), and deep sequenced using the Genome AnalyzerⅡx (Illumina, San Diego, USA) according to the protocol of the manufacturer.

### Sequencing data analysis

The raw sequence data were collected using Illumina’s Sequencing Control Studio software version 2.8 (SCS v 2.8, LC-Bio, USA), and extracted from the image files generated using Illumina Genome Analyzer Pipeline software (Illumina, San Diego, USA). The distribution 15–30 nt sRNA fragments was evaluated, and the poor quality sequences, which included junk sequences, sequences of repetitive bases, as well as dimers and trimers were removed before the sequences were mapped to mRNA (http://www.ncbi.nlm.nih.gov/), RepBase (http://www.girinst.org/repbase), RFam (http://rfam.janelia.org). The remaining data was used to find conserved miRNAs produced by cucumbers in response to CGMMV infection using a BLASTn search of the pre-miRNA (mir) and mature miRNA (miR) sequences listed in the latest release of the miRBase 21.0 (htt://mirbase.org). All of the cucumber sRNA sequences were then used to BLASTn search the cucumber genome database (http://cucumber.genomics.org.cn/). In addition, their secondary hairpin structure was assessed using RNAfold software (http://rna.tbi.univie.ac.at/cgi-bin/RNAfold.cgi) to identify potential miRNA precursor sequences. Before being assigned as miRNAs, each candidate sequence had to fulfill several criteria including being an accurate match to genomic sequences obtained from cucumber. The mature miRNA sequence should be located in arm of hairpin structure and its precursors should also accumulate to a higher level of expression *in vivo*. The minimal folding energy (MFE) and minimal free folding energy indexes (MFEI) as well as other attributes of potential miRNA precursors were then assessed according to the fifteen criteria listed in Table 1 [32–35].

**Table 1:** Criteria used to screen putative miRNAs.

Criterion No.	Content
1	Not contain the adaptor/acceptor in sequencing sequences.
2	Sequences length between 17–25 nt.
3	No-exceed 80% base A, or G, or C, or T.
4	Not match the mRNAs, rRNA and tRNA.
3	Minimal Free energy (dG in kCal/mol) ≤ -15.
5	Minimal folding free energy index (MEFI) ≥ 0.7 *.
6	Number of allowed errors in one bulge in stem ≤ 12.
7	Number of base pair in stem region ≥ 16.
8	Length of hairpin which the up and down stem plus terminal region ≥ 50 nt.
9	Length of terminal loop ≤ 350 base pair.
10	Number of allowed errors in one bulge in mature region ≤ 8.
11	Number of allowed biased errors in one bulge in mature region ≤ 4.
12	Number of allowed biased bulges in mature region ≤ 2.
13	Number of base pair (nt) in mature or mature* region ≥ 12.
14	Percentage of small RNA in stem region (pm) ≥ 80%.
15	Number of allowed errors in mature region ≤ 7.

*Combined with LC science screening criterion and the results of previous studies [27, 28, 32–34].

### Validation of cucumber miRNAs by chip expression profiling

The hybridization chip was prepared using the 349 putative miRNAs identified from the sequencing and screening analysis, including the 8 novel miRNAs and 23 known cucumber miRNAs, as well as 649 sequences from other plant species closely related to the *Cucurbitaceae* (including *Arabidopsis lyrata*, *Arabidopsis thaliana*, *Brassica napus*, *Brassica oleracea*, *Brassica rapa*, *Carica papaya*, *Cucumis melo*, *Gossypium arboretum*, *Gossypium herbaceum*, *Gossypium hirsutum*, *Gossypium raimondii*, *Theobroma cacao*, *Populus euphratica* and *Populus trichocarpa*) in the NCBI database, which were used for comparison. All leaf and flower samples (10, 30, 50 dpi leaves and flowers) were collected in triplicate from three separate plants. Small RNA (5.0 ng) extracted from each sample was used to probe the hybridization chip. The experiment was conducted using a μParaflo microfluidic chip (LC Sciences, Houston, TX) at 34°C. The hybridization images were collected using a laser scanner (GenePix 4000B, Aoxn, USA) and transformed to digital data using the Array-Pro image analysis software (Media Cybernetics, Washington, USA). The signal detection threshold was set to 500 after the background signal had been eliminated. Finally, the hybridization signals were detected and quantified using a cyclic LOWESS filter and subjected to data analysis (Locally-weighted Regression) [36]. Each hybridization was performed twice and Fisher’s least significant difference test was used to detect significant differences at p-value ≤0.01 (signal >500).

### Prediction of target genes associated with conserved miRNAs using gene ontology and Kyoto encyclopedia of genes and genomes analysis

The target gene predictions for the results of chip hybridization analysis were made using miRanda [http://www.microrna.org] and psRNATarget [http://plantgrn.noble.org/psRNATarget/] software. Putative miRNA sequences were then annotated using the cucumber UniGene database hosted at the Cucurbit Genomics Database [http://www.icugi.org] and the CGMMV genome database [http://www.ncbi.nlm.nih.gov], as well as the GO and KEGG databases for *A*. *thaliana* [http://www.arabidopsis.org].

## Results

### Solexa sequencing and analysis of *C*. *sativus* small RNAs

**Fig 2:**
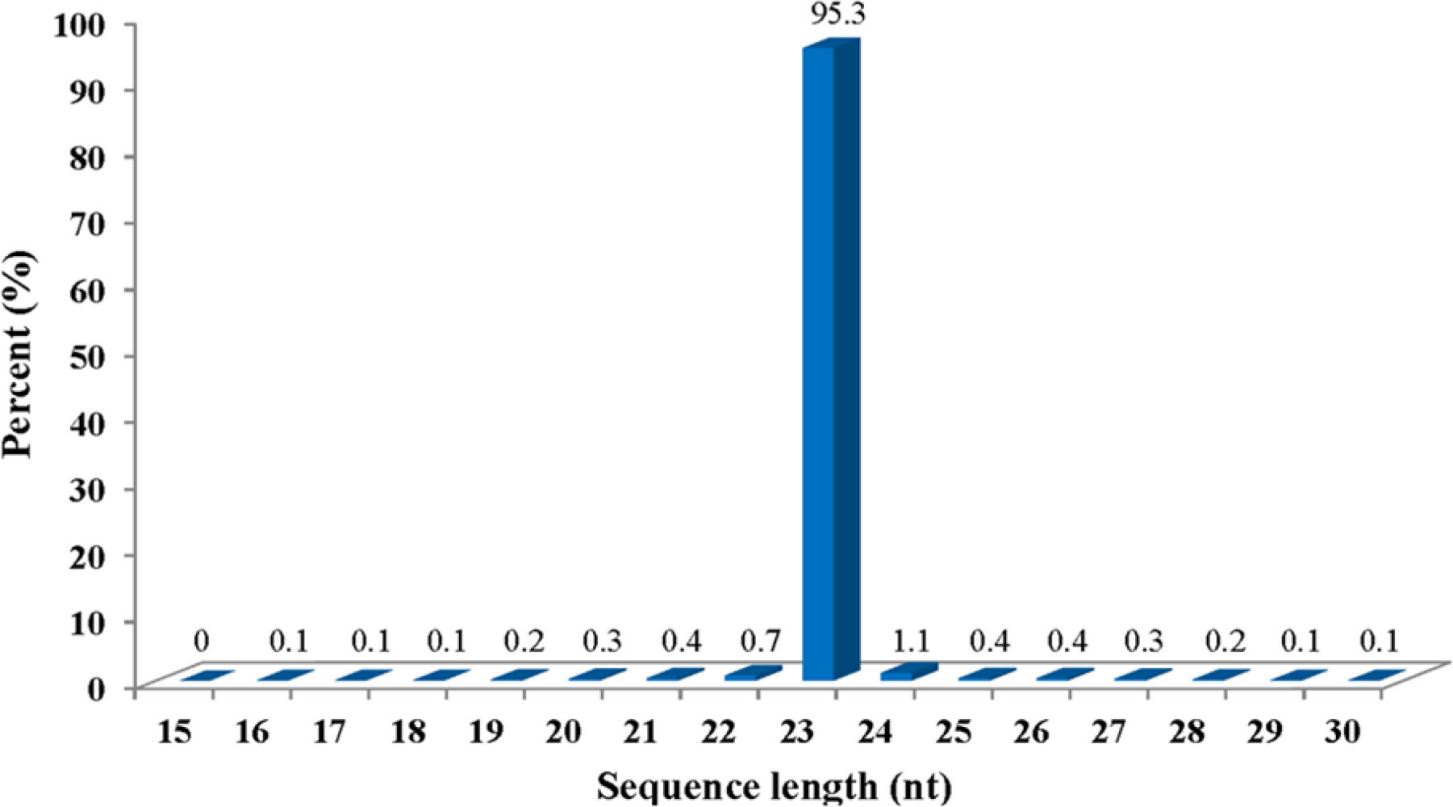
Length distribution of mapped sequence reads from cucumber plants infected with CGMMV.

The cucumber cultivar Zhongnong 16 [31] is one of the most widely cultivated cucumber cultivars in the area around Beijing and was selected for the current study because preliminary inoculation experiments revealed that it was highly sensitive to CGMMV. Leaf samples were collected at three different time points (10, 30 and 50 dpi), which were representative of both the progress of disease development and the lifecycle of the cucumber plants. When kept under greenhouse conditions at 27–32°C with natural light the cucumber plants were found to display typical symptoms of disease, including mottle and mosaic patterning as well as deformity of shape, at 15–30 dpi. The 10 dpi sample was selected to coincide with the asymptomatic period post inoculation, while at 30 dpi and 50 dpi the samples were representative of the symptomatic phase, but also corresponded to the period of vegetative growth at 30 dpi and the transition to reproductive maturity at 50 dpi, at which point the male and female flowers were also collected. The CGMMV status of both the inoculated and non-inoculated samples was confirmed to be CGMMV-positive by SEM and RT-PCR (Fig 1) before sRNA extraction and sequencing. A total of 16,668,516 raw reads were produced. However, after filtering out adapter sequences and junk reads (length <15 nt) according to the criteria of Illuminas’s Genome Analyzer Pipeline software and ACGT101-miR program, this figure was reduced to 16,401,912 mappable reads, which constituted 98.4% of the total sequence population. The mappable sequences were compared with RNA databases including Repbase and RFam to eliminate other RNA species and highlight candidate miRNAs. This process identified 71,154 mappable reads corresponding to mRNA (0.4%), 742 reads of repetitive sequences (ca. 0%), and 50,799 reads corresponding to rRNA, tRNA, snRNA, snoRNA (ca. 0.3%), as well as 1,216,061 no-hit un-mapped reads (7.4%). The putative miRNA sequences were then filtered further according to the FE or MEFI criteria listed in Table 1, which yielded a final total of 15,063,156 mappable reads within the 15–30 nt range, of which 95.3% were 23 nt in length (Fig 2). These results differed to those of a previous study investigating cucumbers infected by *Hop stunt viroid*, in which only 10.40% of the mappable reads were 23 nt in length [27].

### Identification and Characterization of *C*. *sativus* miRNAs

**Fig 3:**
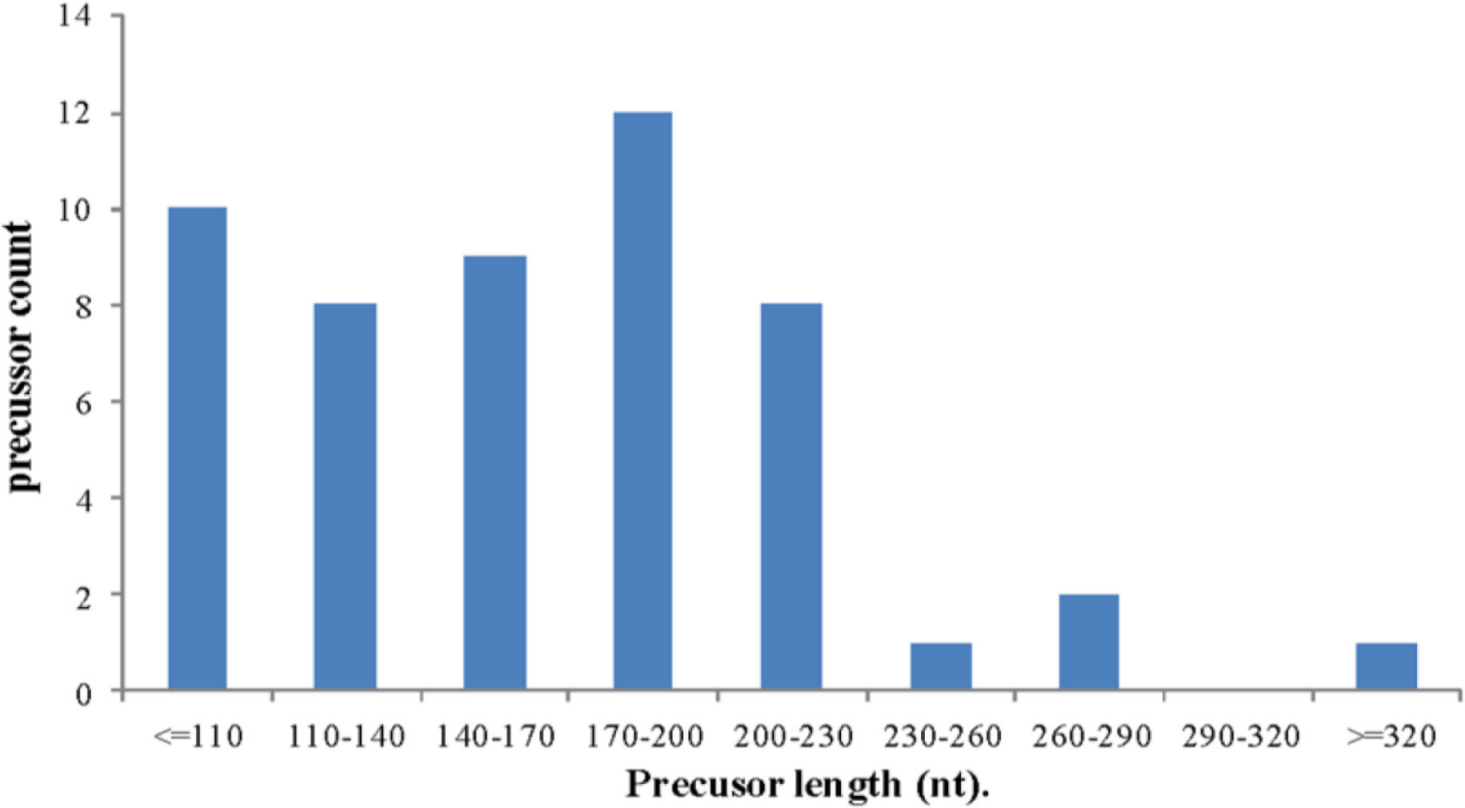
Length distribution of cucumber pre-miRNAs identified in the current study.

**Fig 4:**
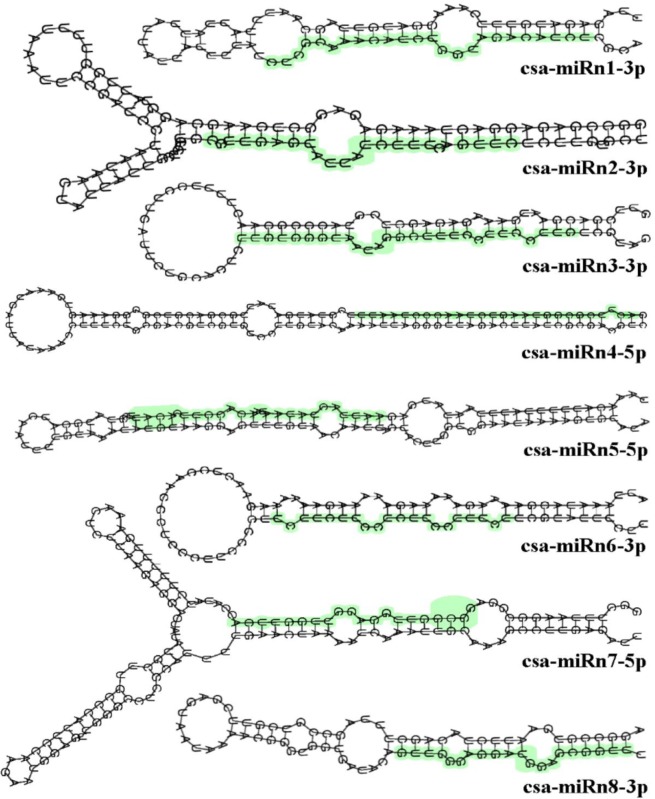
Secondary structure of novel cucumber miRNAs predicted by bioinformatic analysis using RNAfold software (http://rna.tbi.univie.ac.at/cgi-bin/RNAfold.cgi).

The 15,063,156 mappable reads were compared with mirs (Pre-miRNA)/miRs (mature miRNAs) from the genome of cucumber and closely related cucurbitaceae species in the miRBase (http://mirbase.org). The results revealed 23 known cucumber miRNAs that fulfilled the criteria defining mature miRNAs (Tables 1 and 2). The length of their corresponding pre-miRNA sequences ranged from 80–346 nt (Fig 3). The secondary stem-loop hairpin structures of the miRNAs were located in both the 5' and 3' arms, with 9 miRNA sequences being located in the 5' arm and the other 14 in the 3' arm. The average MFEI and MFE values of the miRNAs, which are considered important standards [32], were -2.5 kcal/mol and 1.07, respectively. Given that the secondary structure of pre-miRNA is an important screening criterion to distinguish them from other small RNAs, further predictions regarding the secondary structures of the known miRNAs were made based on existing cucumber miRNAs and genomic data. These results confirmed that the 23 known miRNAs produced in response to CGMMV infection corresponded to mature miRNA (Table 2). Although 8 of the 23 confirmed miRNAs have previously been reported in cucumber [27, 28], the current study represents the first example of the remaining 15 being observed *in vivo*. Furthermore, according to the criteria of the previous studies [27, 28] that were used to compile the strict screening criteria listed in Table 1, there were as many as 120 other predicted candidate (PC) miRNAs (S1 Table, S1 Appendix, S2 Appendix) among the mappable reads that almost fulfill the screening criteria for pre-miRNAs. Alignment of the unmapped sequences with cucumber genomic DNA and filtering according to the screening criteria for miRNAs, indicated that 12 new miRNAs had been isolated. However, analysis of the secondary structure of their precursors, and their location in arm of the stem-loop structure (http://rna.tbi.univie.ac.at/cgi-bin/RNAfold.cgi) indicated that only eight of the 12 could be confirmed as novel miRNAs (S2 Table, Fig 4, S1 Appendix, S2 Appendix). The MFE and MFEI of their pre-miRNAs averaged -32.214 kcal/mol and 0.738, respectively.

**Table 2:** Known miRNAs isolated from cucumber infected with CGMMV.

No.	Name (csa)	Sequence	L (nt)	A	G	C	T	PL (nt)	La	MFE (kcal mol^-1^)	MEFI
1	miR156b	CTCTCTCTCTCTCTCTC	17	0	0	9	8	129	3	-61.3	1.2
2	miR164a	TTCATCTTCATCTTCTT	17	2	0	5	10	156	5	-66.4	0.8
3	miR167h	GAAAGAACAAGAACAATCTTCTAC	24	12	3	5	4	151	3	-53	0.8
4	miR171d	CGCTCTCAAAATGAGCCCACTGCAC	25	7	4	10	4	103	3	-53.8	1
5	miR172c	CTTGATGATCCTGCAGAT	18	4	4	4	6	133	3	-60.5	1.2
6	miR390	CTCATCTCTTTCTCTCTCAAACTCC	25	4	0	11	10	209	3	-78.8	0.8
7	miR395a	CTTTATTGATATATAAT	17	6	1	1	9	110	5	-48.4	1
8	miR437a	TTTTTTGTTGATCTTGCTGGTCG	23	1	6	3	13	190	3	-52.8	1
9	miR536a	CGGAAAGATACCAGAAGATACC	22	10	5	5	2	199	3	-72	0.8
10	miR812f	TCTTATTTAATGTTTTTT	18	3	1	1	13	179	5	-64.7	1.5
11	miR827	GCGAAAATGAACTTTTC	17	6	3	3	5	131	5	-75.5	1.2
12	miR838	CTTTTCTTCTACTTATGCTCATAC	24	4	1	7	12	207	3	-79.9	1
13	miR902a	GTCAAGAGGAAGAAAATTA	19	10	5	1	3	223	3	-69.6	0.7
14	miR902c	GTCAAGAGGAAGAAAATTA	19	10	5	1	3	134	3	-54.2	1
15	miR1082a	CTTTCTCTCTCTCTCTCCT	19	0	0	9	10	160	3	-79.1	0.9
16	miR1310	CCTCGACCTATTCTCATGGAATTC	24	5	3	8	8	80	5	-35.4	0.8
17	miR2087	CATTTTAGTCATTTACTACCCA	22	6	1	6	9	265	3	-157.8	1.5
18	miR2585a	TTCGACCAACTTAATCA	17	6	1	5	5	346	5	-168.8	1.3
19	miR2608	CTTTGTCTCTCTCTCTCTCTT	21	0	1	8	12	212	3	-58.8	0.9
20	miR2673a	CTTCCTCCTCCTCTTCTCCTTCT	23	0	0	12	11	247	5	-71.6	0.7
21	miR3440b	ACTAATACAAAATGTGCCGATT	22	9	3	4	6	125	3	-70.4	1.4
22	miR5015a	CTGTTGTTGTTGCTGTTACTA	21	2	5	3	11	173	5	-88.7	1.6
23	miR5538	ATGAGGAACTACTGAACTCAATCAC	25	10	4	6	5	123	5	-42.9	0.8

*P, Precursor, L, Length, La, Location of arm, MFE, Minimal folding energy, MEFI, Minimal folding free energy index.

### Microarray analysis of miRNAs from cucumbers and closely related plant species, and bioinformatic analysis of their associated target genes

Hybridization chips containing a total of 998 miRNA sequences were used to generate an expression profile to validate the target sequences of miRNAs associated with CGMMV infection. The hybridization chips were prepared using the 349 unique miRNA sequences including the 23 known miRNAs, 8 novel miRNAs, 120 PC cucumber miRNAs, as well as 649 miRNAs (unique sequences) from the miRBase [http://www.mirbase.org/, Release 21, June 2014] corresponding to miRNA sequences from a range of other angiosperm plants including *Arabidopsis thaliana* and *Populus trichocarpa* as well as various other species belong to the orders *Capparales*, *Violales*, *Malvales* and *Salicales*, which all belong to the *Dilleniidae*, the same subclass as cucumber. Of particular interest were four homologous miRNAs, 3 from *C*. *sativus* (*Cucurbitaceae*) and 1 from *Carica papaya* (*Caricaceae*), which had been identified in a previous study. The chips were probed with sRNA extracted from the leaves and flowers of cucumbers at different time points post inoculation with CGMMV, as well as with the non-inoculated controls.

**Fig 5:**
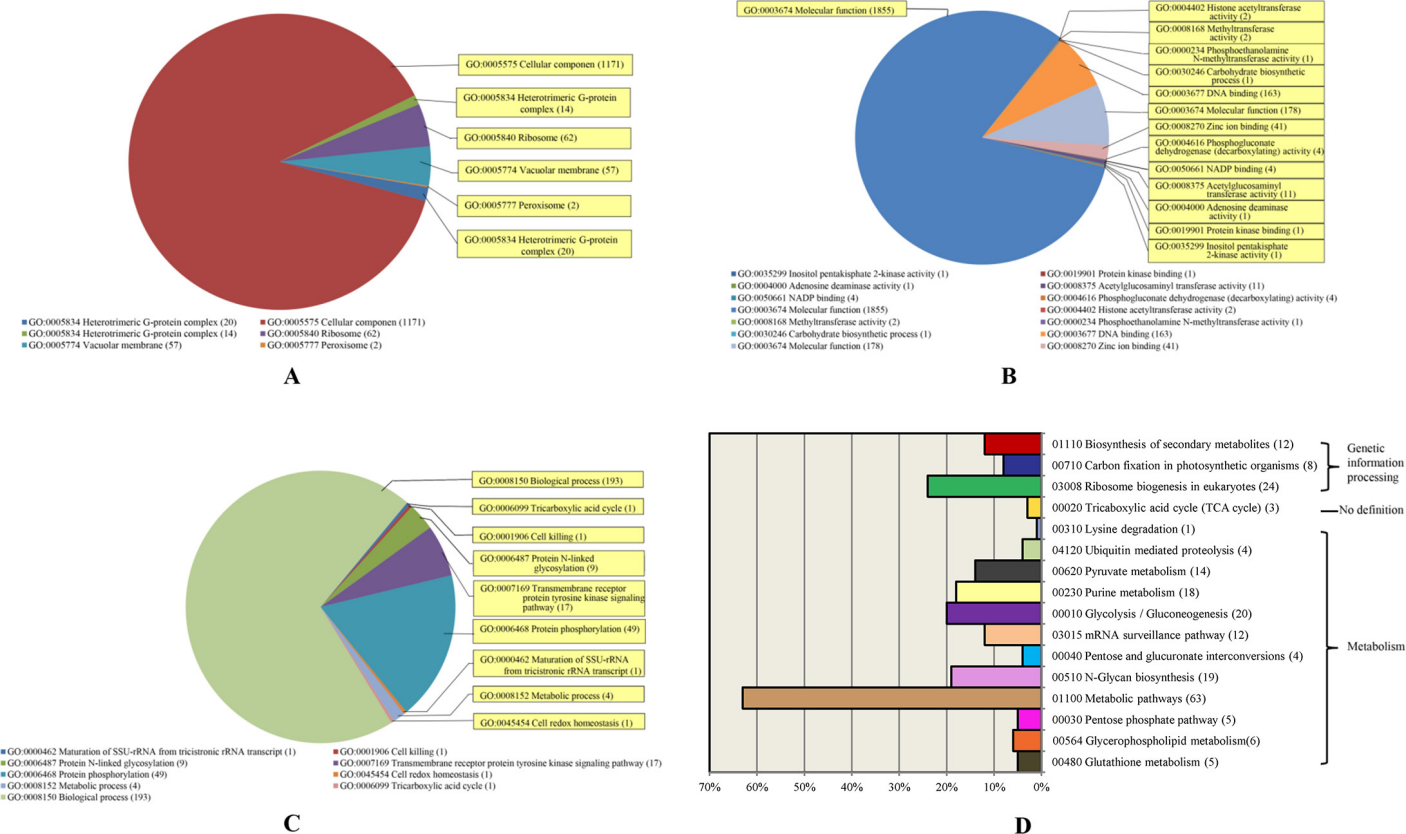
Overview of the microarray analysis. Chips prepared with miRNAs from cucumber and conserved miRNAs from species closely related to cucumber were probed with sRNA extracted from the leaves and flowers of cucumbers at different time points post inoculation with CGMMV. A, B and C, The proportions of genes annotated by GO analysis in three categories: molecular function, biological process and cellular component, respectively. D. Results from the KEGG analysis, which classified the target genes into different functional pathways [37, 38].

The analysis identified 82 conserved miRNAs, similar to the miRNAs produced in the closely related plant species as described as above, 3 putative miRNAs and 3 novel miRNAs that were expressed in CGMMV-infected leaves and flowers (S2 and S3 Tables). The target genes of the conserved miRNAs were further investigated by cluster analysis using Cluster and Treeview software (P-value of Fisher’s exact test < 0.01). The results indicated that the expression level of 3867 related genes could be affected by the regulatory functions of the cucumber miRNAs. Gene ontology annotation indicated that 2265 of these genes were involved in molecular function, 1326 as cellular components and 276 in biological process (Fig 5A, 5B and 5C). The subsequent KEGG analysis (P-value of Fisher’s exact test <0.1) showed that the annotated genes were involved in 16 pathways, which could be classified into three groups including metabolism, genetic information processing, and those with no definition comprising 63 related pathways, including glycolysis, gluconeogenesis and the tricarboxylic acid cycle (TCA cycle). More than 80% of the predicted target genes were involved in metabolic pathways and ribosome biogenesis. The remaining genes were associated with the biosynthesis of secondary metabolites, carbon fixation in photosynthetic organisms, the TCA cycle, lysine degradation, ubiquitin mediated proteolysis, pyruvate metabolism, purine metabolism, glycolysis/gluconeogenesis, mRNA surveillance pathways, pentose and glucuronate interconversion, N-glycan biosynthesis, the pentose phosphate pathway, glycerophospholipid metabolism and glutathione metabolism (Fig 5D).

The GO and KEGG pathway analysis also predicted many target genes associated with the miRNAs that were produced by cucumbers in response to CGMMV infection. Genes with functions related to disease symptoms and resistance were of particular interest because they provide clues relating to potential pathogenic mechanisms and resistance genes. Comparison of the hybridization signals produced by the infected and non-infected samples at different time points post-inoculation revealed that different miRNAs were typically expressed at different developmental stages (S3 Table), with 22, 27, 13, 14 and 1 individual miRNAs being expressed in the leaves at 10, 30, 50 dpi, and in the male (mfi) and female (ffi) flower samples, respectively (p-value <0.01, hybrid signal >500). However, when the signal was <500, the number of miRNAs for the five samples were 14, 19, 11, 3 and 1, respectively. When the results produced under both sets of parameters were combined a total of 88 validated miRNAs had been identified, which represented 47 different miRNAs families, including 16 already documented in cucumber [27, 28], as well as 3 putative miRNA families and 3 novel miRNAs not previously confirmed *in vivo*. The remaining 25 families, previously characterized in closely related species, are described in cucumber for the first time in the current study. The precursor sequences of the 88 cucumber miRNAs were subsequently used to blast search the cucumber genome [http://www.icugi.org/cgi-bin/ICuGI/genome/index.cgi] in an attempt to identify genes associated with pathogenicity.

## Discussion

The current study generated a total of 15,063,156 mappable reads. Unexpectedly, the vast majority of them (95.3%) were shorter in the case of cucumber and wheat studies at 23 nt rather than 24 nt, but longer than the sRNAs of tomato which were 21–22 nt [9]. Moreover, only 1.1% of the sRNAs recovered from cucumbers infected with CGMMV were 24 nt, which is a huge difference compared with previous studies. Both of healthy cucumber leaves and roots, in which 24 nt sRNAs were the most abundant (>40%) [28]. Although the anomalous results could have resulted from differences in the sequencing procedures, it is also possible that the high proportion of 23 nt sRNA could have been influenced by CGMMV infection itself. Variation in sRNA length has also been observed in previous studies of cucumber including cucumber leaves and phloem infected with *Hop stunt viroid* [27], healthy cucumber leaves and roots [28], and leaves and roots from grafted cucumbers and pumpkins [39], and it is possible that biotic factors such as CGMMV infection as well as other abiotic factors could affect the proportion of sRNAs of different lengths.

Analysis of the mapped reads revealed there to be 23 known cucumber miRNAs as well as a further 120 PC miRNAs. The 23 known cucumber miRNAs appeared to be produced at particular time points in the leaf samples, with 12, 8 and 3 miRNAs being identified at 10, 30 and 50 dpi, respectively, and none being recovered from the flower samples. The functions of many plant miRNAs have been determined and released in the miRBase, which was a useful resource for determining the predicated target genes of these miRNAs. One of the most interesting of the miRNAs produced at 10 dpi was miR156, which was first isolated from *Theobroma cacao* [40] and is conserved in *Cucumis melo* [41], a close relative of cucumber. Previous studies of these homologues have shown miRNA156 to be associated with 11 members of the SQUAMOSA promoter binding protein like (SPL) family, which can cause altered carotenoid levels in seeds *via* the suppression of SPL genes [42]. Previous research has also demonstrated that SPL genes influence leaf morphogenesis in *Arabidopsis thaliana* [43]. The bioinformatics analysis in the current study predicted that miR156 regulates the target genes Csa6M091970.2 and Csa6M091970.1 (http://www.icugi.org/cgi-bin/ICuGI/index.cgi), proteins that have been shown to play a role in many metabolic processes including microbial defense, signal transduction, regulation of cell development, and pollen maturation [44–46]. In addition, miR156 can also regulate Csa5M198140.1 and Csa1M038340.1, which can affect the development of leaves, flowers, fruit and lateral roots. It has proofed that miR156-mediated age pathway can regulate flowering time in many species including *Arabis alpina*, *Solanum tuberosum* (cv. ssp. *andigena* 7540) and *Panicum virgatum* [47]. It is therefore possible that miR156 is responsible for the deformation of leaves and fruits observed during CGMMV infection as well as the delayed blooming and reduced fruit production. Previous research identified that miR156 can notably affect biomass production in *Panicum virgatum* and *Medicago sativa*, respectively [48, 49]. These results strongly suggest that miR156 can play roles with production of cucumber. Another interesting miRNA active at 10 dpi was mi390. Previous studies in *Physcomitrella paten* have shown that miR156 and miR390 can affect developmental timing. In particular it was found that the over expression of miR390 resulted in the delayed formation of gametophores [50]. It is therefore possible that the expression of miR390 during CGMMV-infection could delay flowering and affect fruit production in cucumbers.

Two of the miRNAs active at 30 dpi, miR171c and miR172d, which have previously been associated with plant development including the regulation of transcription and growth, phloem transport and the differentiation and development of flower organs, were predicted to target several genes including Csa6M109640.1, Csa3M020600.1, Csa6M296960.2 and Csa6M296960.1. Previous research has indicated that these genes probably have a role in the transport of virus particles within infected cucumber plants [51]. Moreover, it has also been demonstrated that these genes mainly regulate flower development and organ morphogenesis [52]. For example, allelic mutation experiments have shown that the transcription factors Csa6M296960.2 and Csa6M296960.1 (*APETALA1/2*, *AP1/2*) can control the formation of sepals in *Arabidopsis* [53]. It is also interesting to note that Csa1M605660.1, which revealed in the cucurbit genomics database annotation (http://www.icugi.org/cgi-bin/ICuGI/tool/blast.cgi), is a target gene for miR2673 at 30 dpi, and associated with the growth of the pollen tube. Taken together these results indicate that the miRNAs highlighted by the current study could be of great significance in elucidating the factors influencing the delayed flowering and reduced fruit production observed in CGMMV-infected cucumbers and that they therefore warrant further study.

The target genes of the 8 novel miRNAs (S2 Table) were also assessed. Two of the novel miRNAs, csa-miRn1-3p and csa-miRn2-3p, were found to affect the transcription factors Csa1M109320.1 and Csa6M056520.1, respectively. Csa1M109320.1 is an example of a myeloblastosis (MYB) transcription factor, which contains a 52 amino acid region that binds sequence-specific regions of DNA and play a role in the regulation of many metabolic processes in plants, including cellular morphogenesis and development, secondary metabolism and responses to biotic and abiotic stresses, meristem formation and the cell cycle [54, 55]. In contrast, Csa6M056520.1 is a transcription factor containing the basic leucine zipper domain (bZIP). Evolutionary analysis has shown that bZIP transcription factors occur in all plants as a consequence of sharing a common ancestor, and are important in light and stress signaling, and flower development [56, 57]. In addition to their other functions, it is known that both MYB and bZIP transcription factors have roles during pathogen infection. Previous studies have shown that MYB genes are induced in tobacco plants in response to TMV infection, being important in the hypersensitive response and systemic acquired resistance, while bZIP factors, which bind to the G-Box of the soybean (cv. *Glycine max*) *Chs*15 promoter are also activated during the plant defense response [58–62]. Experimental evidence has also shown that Csa7M073450.1, which is regulated by csa-miRn1-3p, can induce UDP-glycosyltransferase and scopoletin glucosyltransferase (S2 Table), both of which have been associated with pathogen resistance. For example, UDP-glycosyltransferase is necessary for resistance to *Pseudomonas syringae* pv *tomato* in *Arabidopsis* [63] while scopoletin glucosyltransferase can cause *Nicotiana tabacum* to generate precocious lesions during tobacco mosaic virus infection [64].

Two of the other novel miRNA were predicted to be involved in aspects of cell wall metabolism. In this case csa-miRn3-3p was found to target Csa4M578870.1, which encodes a microfibril-associated protein, consisting of glycoproteins. This protein is an essential component of the cell wall and cytoskeleton [65]. The development of the plant cell wall plays crucial roles in plant growth, cell differentiation as well as in response to invading microbes, and it has been suggested that Csa4M578870.1 is associated with the dwarfed appearance of CGMMV-infected cucumber plants [66]. In contrast, csa-miRn5-5p was found to target Csa4M056510.1 (pectin acetylesterase, PAE), which can induce plant cell wall degradation [67], and another regulatory gene Csa2M033380.1 (actin binding/cytoskeleton) associated with the regulation of cytoskeletal microfilaments [68]. Actually, the novel miRNAs csa-miRn6-3p, csa-miRn7-5p and csa-miRn8-3p (S2 Table) has been identified by chip expression profiles, it has display as PC-3p-73705, PC-5p-12288 and PC-3p-64329, respectively (S3 Table). Another of the novel miRNAs csa-miRn6-3p, which was validated by the microarray experiment, was found to regulate many genes including Csa2M033380.1, Csa7M219220.1, Csa3M683670.1, Csa3M117970.1, Csa3M019980.1, Csa2M380020.2, Csa3M119700.1, Csa4M000700.1 and Csa1M424880.1. These genes appear to be involved in lipid biosynthetic/metabolic processes, acyltransferase activity and zinc ion binding, as well as encoding constituents of the thylakoid membrane (http://www.icugi.org/cgi-bin/ICuGI/tool/blast.cgi). Thylakoids have been shown to be associated with phosphorylation and photosynthetic electron transport [69], and as an integral component of the chloroplast it is likely that changes that affect their function could contribute to the symptoms of CGMMV infection such as the mottling of leaves. Another of the novel miRNAs csa-miRn8-3p, was predicted to effect five target genes including a nitrate transporter (Csa7M257340.1), which has previously been associated with crop yield [70]. The target genes of the other two novel miRNAs csa-miRn4-5p and csa-miRn7-5p (S2 Table) could not be determined since the cucurbit genomics database has no description for them.

Previous research has demonstrated that viral infection can alter the molecular function, biological processes and cellular components of host plants [71]. The response of cucumber to viral stress is complex and involves many genes and molecular mechanisms, operating at both the transcriptional and post-transcriptional level. It is possible that miRNAs have important roles in regulating the function of such target genes. The chip expression profiles produced in the current study identified 82 conserved miRNAs, and validated 3 of the putative miRNAs and 3 of the novel miRNAs identified in the initial screening. The microarray analysis also provided valuable data regarding the importance of the different miRNAs at different developmental stages and in different organs (S3 Table). It was hoped that such data would increase our understanding of the host-virus interaction with regard to both the pathogenic mechanism of the virus and the resistance response of the host. It is possible that the changes that occur to host miRNA are related to the life cycle of the virus [72], which regulates host metabolism and its intracellular environment [73]. Bioinformatic analysis of the target genes for the 88 miRNAs expressed in the microarray analysis indicated that a range of biochemical processes were affected by CGMMV infection including DNA or protein kinase binding, cell death and immune responses, all of which can also interact with other miRNA-mediated regulatory networks. For example, the signaling pathway of the transmembrane receptor protein tyrosine kinase (S3 Table, GO:0007169) produced at 10 dpi could influence the ontologically conserved family of miRNAs that include miR156, miR172, miR408 and miR444 and thereby regulate a range of target genes including Csa6M296960.2, Csa6M296960.1, Csa6M091970.2, Csa6M091970.1, Csa6M324830.1, Csa3M168380.1, Csa5M198140.1, Csa3M119480.2, Csa3M119480.1, Csa6M384060.1, Csa3M585890.1, Csa2M171930.1, and Csa5M173540.1. Similarly the heterotrimeric G-protein complex (GO: 0005834) could also play a role in signal transduction by combining their cognate receptors and effectors [74]. Indeed, a recent study using Gα, Gβ and Gγ subunits [75] has demonstrated that heterotrimeric G-protein can play a critical role in the resistance of *Arabidopsis* to *Pseudomonas syringae* infection. The current study identified three miRNAs, miR408, miR160 and miR169, which could affect the expression level of heterotrimeric G-protein at 50 dpi. Furthermore, it was also noted that miR2936, which is known to be specifically expressed in the mature pollen of *A*. *thaliana* [76, 77], targeting an F-box protein encoding genes during floral transition and seed development [78], and miR3638, which was isolated from *Vitis vinifera* [79] produced strong hybridization signals in the flowers of CGMMV-infected plants (p-value <0.01, hybridization signal <500). It is therefore possible that the over expression of both miR2936 and miR3638 in CGMMV-infected could account for the altered flowering time and seed formation observed in diseased cucumber plants. It indicated that the yields reduced of cucumber plants contaminated with CGMMV probably indirect to associate with those two miRNAs. Previous research has also demonstrated that over expression of the F-box domain proteins MAIF1 can reduce abiotic stress tolerance and promote root growth in rice, regulating rice growth and development [80]. Despite the potential importance of these kinds of miRNAs, none of the miRNAs typically associated with cucumber flowers produced significant hybridization signals (>500, p-value <0.01) and it is possible that CGMMV infection could cause them to have reduced expression. MiR319 has been demonstrated to target TEOSINTE BRANCHED/CYCLOIDEA/PCF (TCP) transcription factors involved in multiple development pathways, the activity of TCP can immediate impact on leaf cell number and subsequent to suppress cell proliferation. Finally, affect morphological and size of leaf. Actually, miR396 which represses Growth-Regulating Factor (GRF) transcription factor also come with miR319 to play effects [81], and it also play a critical role for petal growth and development in Arabidopsis [82]. Those results were shown that both miR319 and miR396 were probably associated with leaf malformation and plants dwarf, then finally leading to yield losses.

None of the miRNAs identified in the current study corresponded to previously reported viral-responsive miRNAs, which is probably a consequence of the limited number of viral-responsive miRNAs that have been identified for CGMMV. Despite this, it is interesting to note that 10 families of miRNA that were typically expressed in the infected cucumbers were similar to those found in previous studies of CGMMV-infected plants. Perhaps this is not surprising given that miRNAs are evolutionarily highly conserved and affect the same target genes in different species [27]. Moreover, it is possible that all of the target genes associated with the twenty-five conserved miRNA families, 3 putative miRNAs and 3 novel miRNAs validated by the microarray analysis could be expressed in response to CGMMV infection. It is well established that when plants experience exogenous stress, either biotic or abiotic, their response-related gene expression is significantly altered [83, 84]. The results of the microarray analysis conducted in the current study showed that many of the genes affected by CGMMV infection have similar functions (S3 Table) including inositol pentaphosphate 2-kinase activity (Gene ID: 398771727), cell death (Gene ID: 398753331), transcription factors such as protein kinase binding (Gene ID: 398759683), zinc/magnesium ion binding (Gene ID: 398756973/398774069), resistance related genes such as peroxisome (Gene ID: 398753922) and methyltransferase activity (Gene ID: 398762381). The KEGG analysis showed that the altered expression of these genes in cucumber played important roles in genetic information processing, the biosynthesis of secondary metabolites and metabolic pathways (Fig 5D). Among the 16 pathways highlighted by the KEGG analysis (S5 Table), were 5 genes associated with glutathione metabolism (Pathway ID: 480). Glutathione is an important intracellular antioxidant having roles combatting both abiotic and biotic stress and is known to be closely related to infection by plant pathogens [85–87]. Furthermore, previous studies have demonstrated that alterations in glutathione metabolism can cause the chlorophyll content of leaves to change, further disrupting photosynthesis and it is therefore possible that this is the mechanism by which the leaves and fruits of cucumber plants exhibit mosaic or mottle symptoms when infected with CGMMV [88]. Both the GO and KEGG analysis also showed that the target genes corresponding to the miRNAs expressed in the microarray analysis could be associated with CGMMV infection (S4 and S5 Tables). The GO annotation classified the target genes into 3 categories MF, BP and CC, which had different levels of abundance (MF > CC > BP). Moreover, the results obtained from the hybridization chips indicated that the typical expression of miRNAs varied at different developmental stages and in different organs. The analyses indicated that the number of miRNAs typically expressed in cucumber in response to CGMMV infection changed as the infection progressed. It was particularly interesting to note that the GO analysis predicted that the greatest variety of miRNAs were expressed in leaves at 30 dpi, but that the KEGG analysis indicated that the greatest effect on metabolic pathways occurred at 10 dpi, even though a lower number of miRNAs were involved (S3 Table).

In summary, the current study identified a broad range of miRNAs associated with CGMMV infection of cucumbers including 8 novel miRNAs, 23 previously known Csa-miRNAs, 82 conserved miRNAs, 3 putative miRNAs and 120 PC miRNAs (Table 2, S1–S3 Tables, S1 Appendix, S2 Appendix), many of which were validated by chip hybridization. Bioinformatic analysis and gene annotations were used to predicate the functions of their target genes, which indicated that many were involved in or mediated the regulation of physiological mechanism in cucumber including pathogenesis-related genes associated with the symptom and characteristic growth and development of cucumber infected with CGMMV. It is hoped that further investigation of the miRNAs and target genes implicated in this study could lead to the development of disease-resistant plants.

## Supporting Information

S1 Appendix The sequences and its corresponding second structure of 8 novel and 120 predicated candidate miRNAs. (XLS)

S2 Appendix The structure of 8 novel and 120 predicate candidate miRNAs. (DOC)

S1 Table Predicated candidate miRNAs isolated by high throughput screening that can be mapped to the cucumber genome. (DOC)

S2 Table Novel cucumber miRNAs identified by high-throughput sequencing. (DOCX)

S3 Table Expression of 88 conserved miRNAs in different organs and at different time points post inoculation with CGMMV. (DOC)

S4 Table GO analysis indicating the biochemical roles (p <0.01) for the target genes of the 88 conserved miRNAs identified by hybridization analysis of CGMMV infected cucumbers using miRNA sequences from closely related plant species. (DOC)

S5 Table KEGG analysis identifying biochemical pathways (p <0.1) for the target genes of the 88 conserved miRNAs identified by hybridization analysis of CGMMV infected cucumbers using miRNA sequences from closely related plant species. (DOC)
