# Supplementary material for: High-Throughput Sequencing Identifies Novel and Conserved Cucumber (Cucumis sativus L.) microRNAs in Response to Cucumber Green Mottle Mosaic Virus Infection
Source: PLoS One. 2015 Jun 15;10(6):e0129002. doi: 10.1371/journal.pone.0129002 (PMC4468104; doi:10.1371/journal.pone.0129002)
Supplement: S2 Appendix — (DOC) [file pone.0129002.s002.doc]

The second structure of 8 novel and 120 predicated candidate miRNAs, isolated from cucumber (*Cucumis sativus* L., cv ‘Zhongnong 16’) in response to cucumber green mottle mosaic virus infection.

Authors: H.W Liu, L.X Luo, J.Q Li

Unit: Dept. of Plant Pathology, China Agricultural University, Beijing, P.R. China. 100193.

Contact information: [Huawei.liu@ars.usda.gov](mailto:Huawei.liu@ars.usda.gov).

**The second structure of 8 novel miRNAs.**

1. miRNA name: csa-miRn1-3p, got it by deep sequencing.


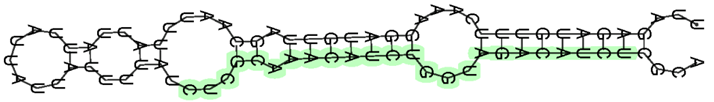


2. miRNA name: csa-miRn2-3p, got it by deep sequencing.


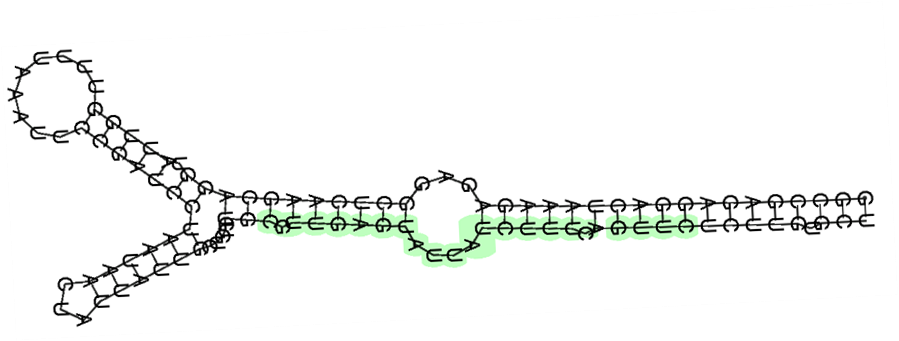


3. miRNA name: csa-miRn3-3p, got it by deep sequencing.


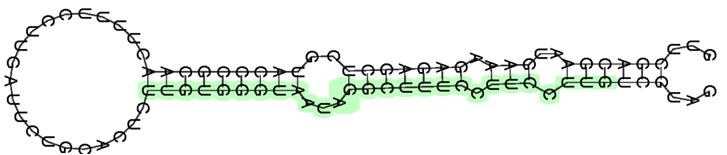


4. miRNA name: csa-miRn4-5p, got it by deep sequencing.


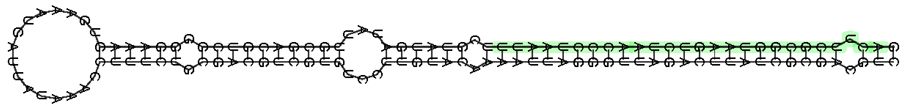


5. miRNA name: csa-miRn5-5p, got it by deep sequencing.


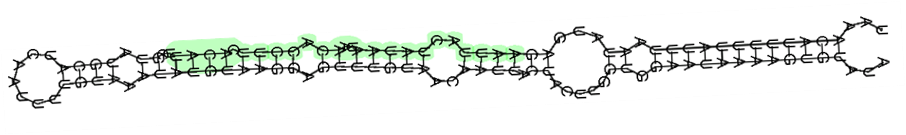


6. miRNA name: csa-miRn6-3p, got it by deep sequencing and experiment validated.


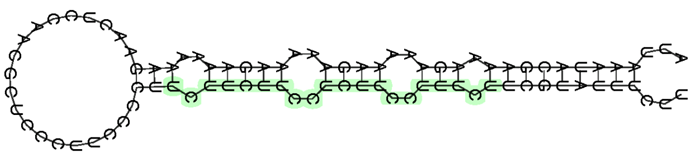


7. miRNA name: csa-miRn7-5p, got it by deep sequencing and experiment validated.


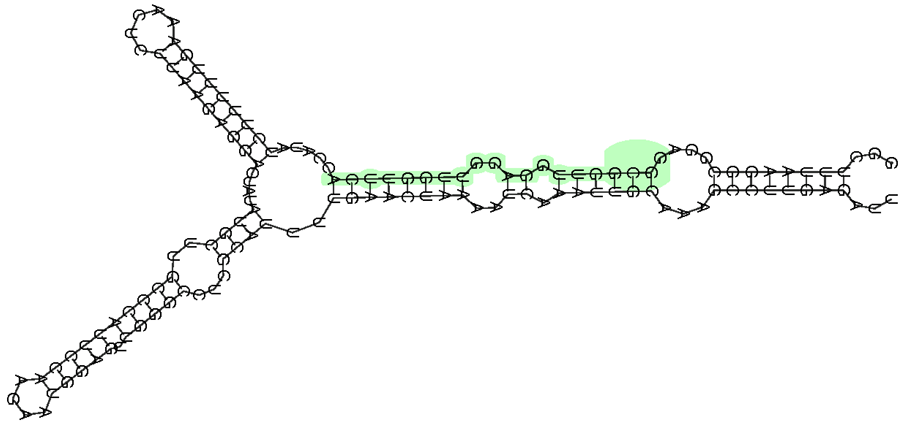


8. miRNA name: csa-miRn8-3p, got it by deep sequencing and experiment validated.


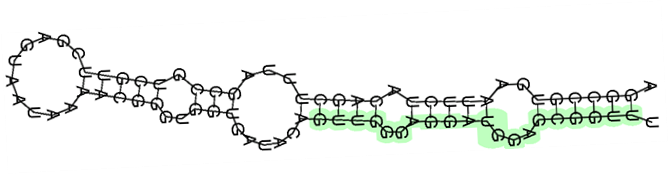


**The second structure of 120 predicated candidate miRNAs.**

1. miRNA name: PC-3p-3557, got it by deep sequencing and no experiment validated.


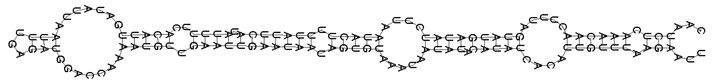


2. miRNA name: PC-3p-4681, got it by deep sequencing and no experiment validated.


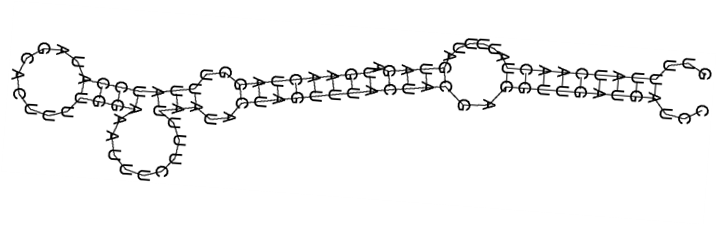


3. miRNA name: PC-3p-8322, got it by deep sequencing and no experiment validated.

**
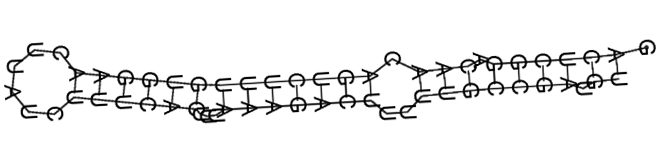
**

4. miRNA name: PC-3p-20844, got it by deep sequencing and no experiment validated.


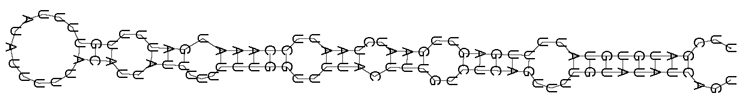


5. miRNA name: PC-3p-25396, got it by deep sequencing and no experiment validated.


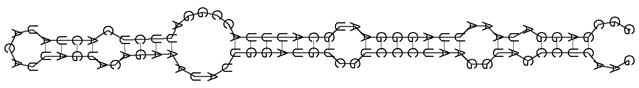


6. miRNA name: PC-3p-28211, got it by deep sequencing and no experiment validated.


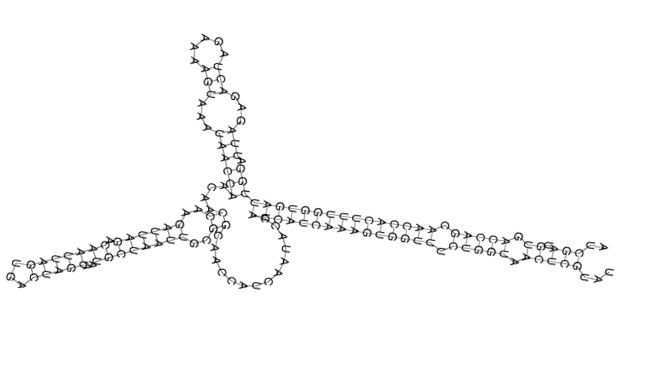


7. miRNA name: PC-3p-31277, got it by deep sequencing and no experiment validated.


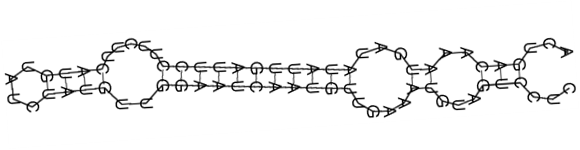


8. miRNA name: PC-3p-34277, got it by deep sequencing and no experiment validated.


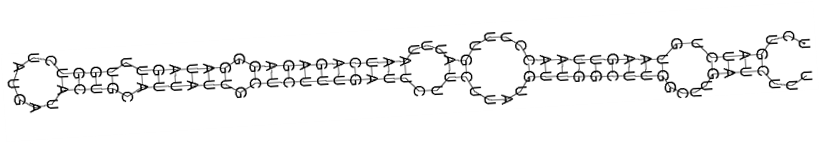


9. miRNA name: PC-3p-37117, got it by deep sequencing and no experiment validated.


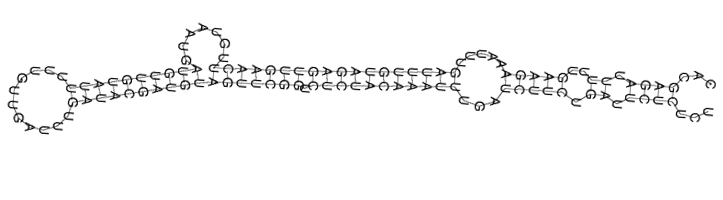


10. miRNA name: PC-3p-37565, got it by deep sequencing and no experiment validated.


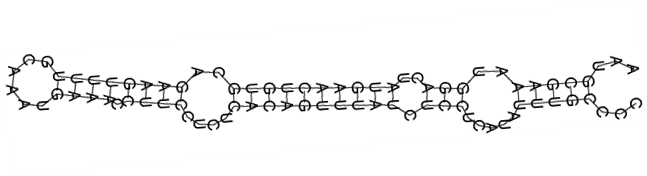


11. miRNA name: PC-3p-38361, got it by deep sequencing and no experiment validated.


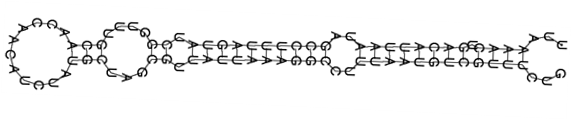


12. miRNA name: PC-3p-39976, got it by deep sequencing and no experiment validated.


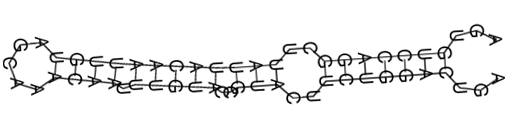


13. miRNA name: PC-3p-41177, got it by deep sequencing and no experiment validated.


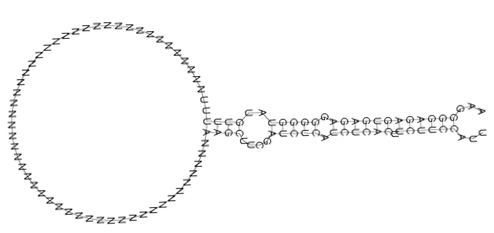


14. miRNA name: PC-5p-44253, got it by deep sequencing and no experiment validated.


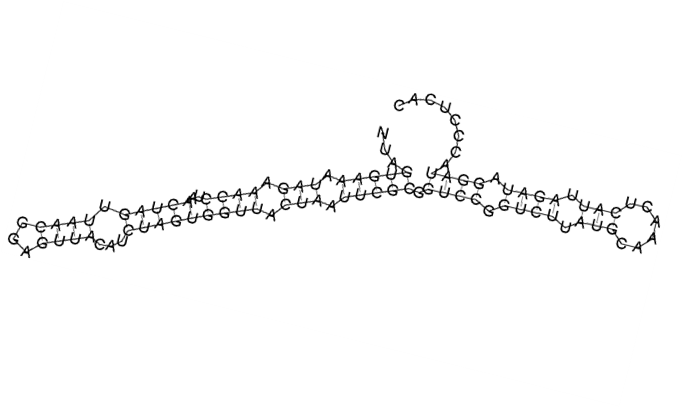


15. miRNA name: PC-3p-45338, got it by deep sequencing and no experiment validated.


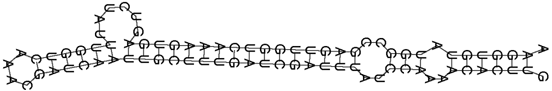


16. miRNA name: PC-3p-46648, got it by deep sequencing and no experiment validated.


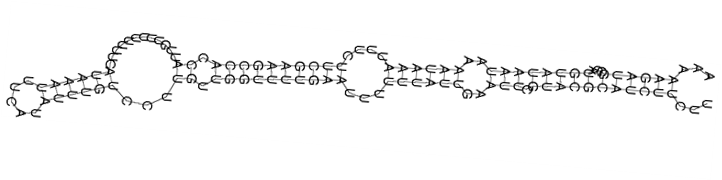


17. miRNA name: PC-3p-47140, got it by deep sequencing and no experiment validated.


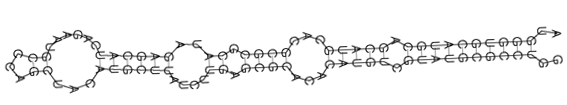


18. miRNA name: PC-3p-48988, got it by deep sequencing and no experiment validated.


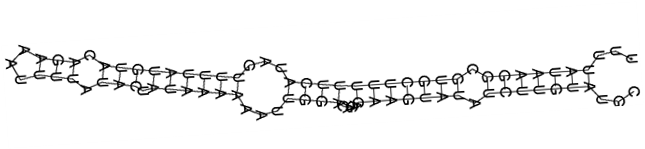


19. miRNA name: PC-3p-50340, got it by deep sequencing and no experiment validated.


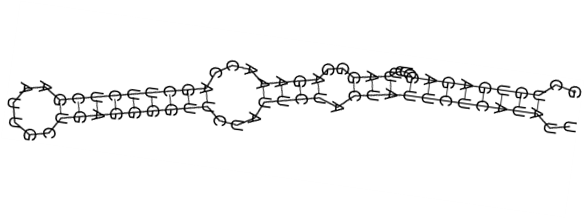


20. miRNA name: PC-3p-50452, got it by deep sequencing and no experiment validated.


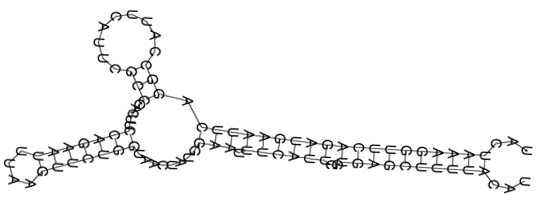


21. miRNA name: PC-3p-50621, got it by deep sequencing and no experiment validated.


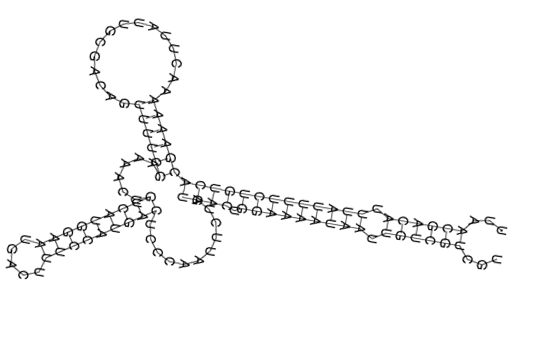


22. miRNA name: PC-3p-50720, got it by deep sequencing and no experiment validated.


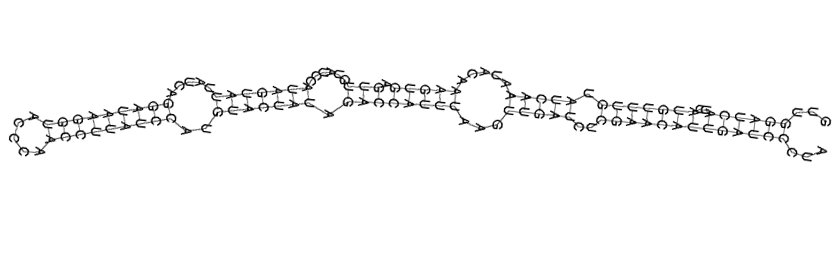


23. miRNA name: PC-3p-51738, got it by deep sequencing and no experiment validated.


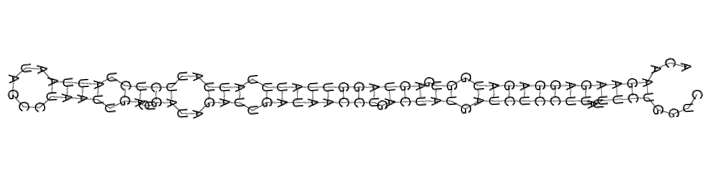


24. miRNA name: PC-3p-59087, got it by deep sequencing and no experiment validated.


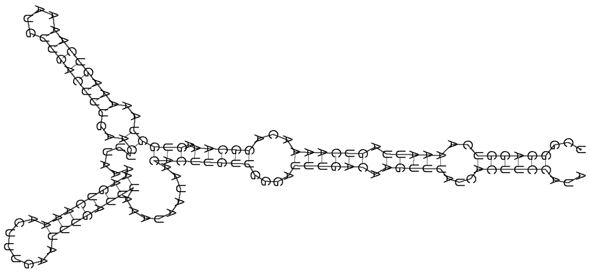


25. miRNA name: PC-3p-59313, got it by deep sequencing and no experiment validated.


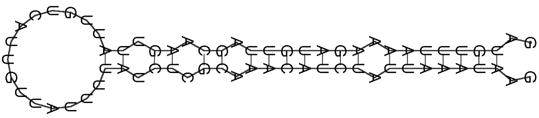


26. miRNA name: PC-3p-67700, got it by deep sequencing and no experiment validated.


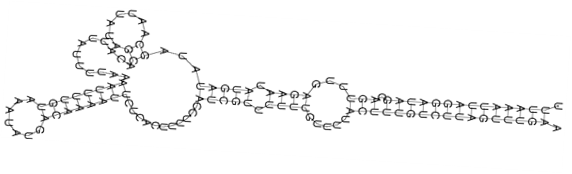


27. miRNA name: PC-3p-69764, got it by deep sequencing and no experiment validated.


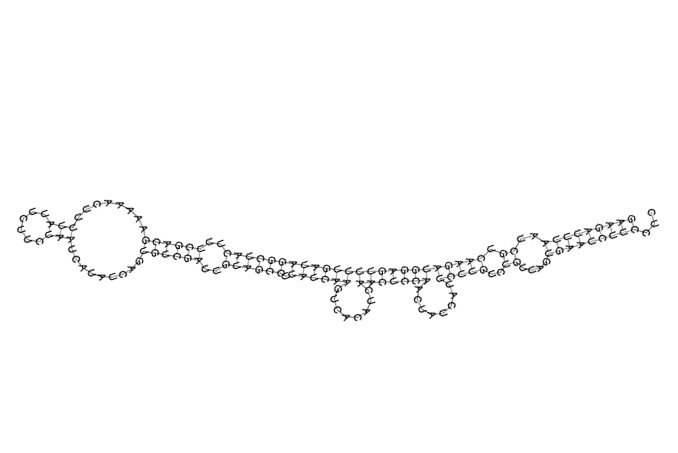


28. miRNA name: PC-3p-70101, got it by deep sequencing and no experiment validated.


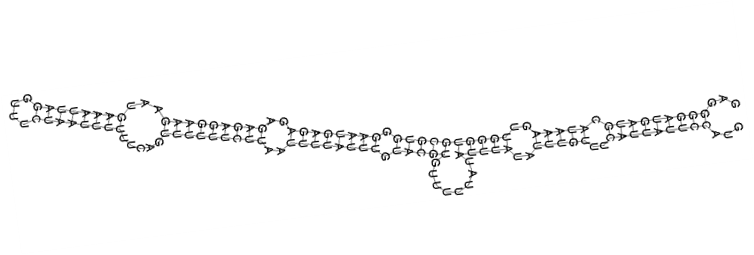


29. miRNA name: PC-3p-70771, got it by deep sequencing and no experiment validated.


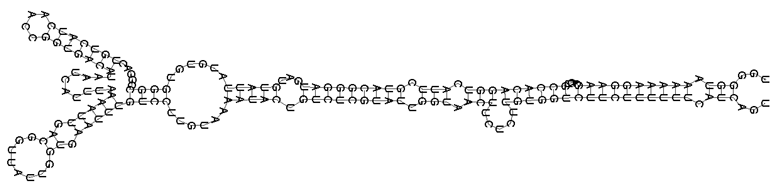


30. miRNA name: PC-3p-71762, got it by deep sequencing and no experiment validated.


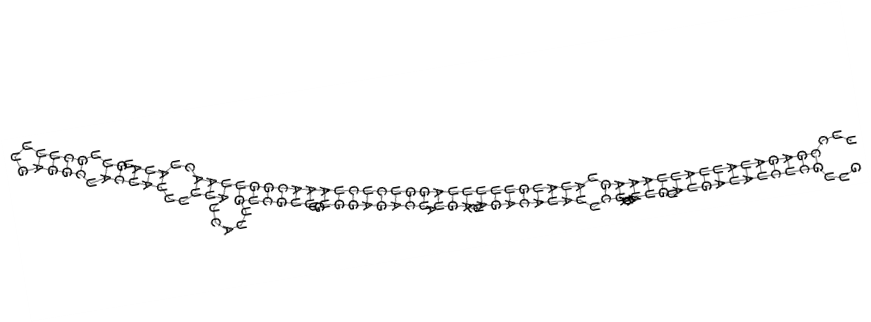


31. miRNA name: PC-3p-72026, got it by deep sequencing and no experiment validated.


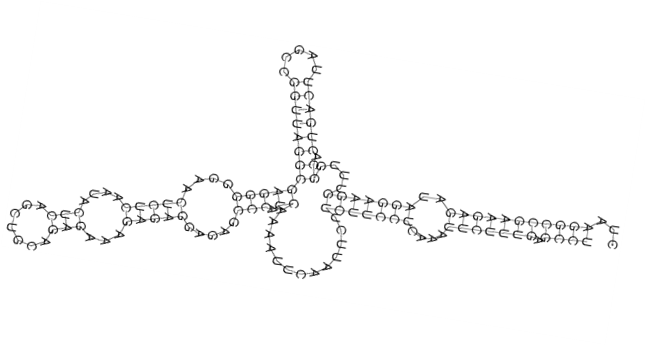


32. miRNA name: PC-3p-76546, got it by deep sequencing and no experiment validated.


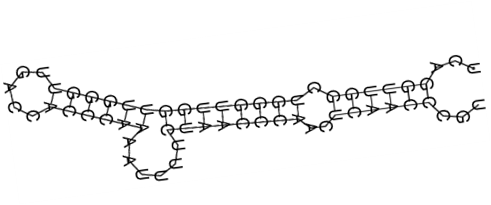


33. miRNA name: PC-3p-87233, got it by deep sequencing and no experiment validated.


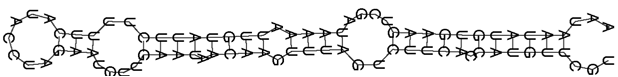


34. miRNA name: PC-3p-87931, got it by deep sequencing and no experiment validated.


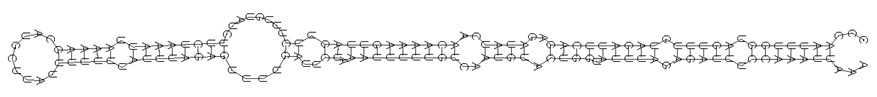


35. miRNA name: PC-3p-88917, got it by deep sequencing and no experiment validated.


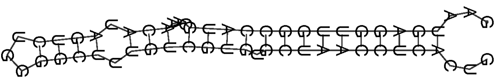


36. miRNA name: PC-3p-95926, got it by deep sequencing and no experiment validated.


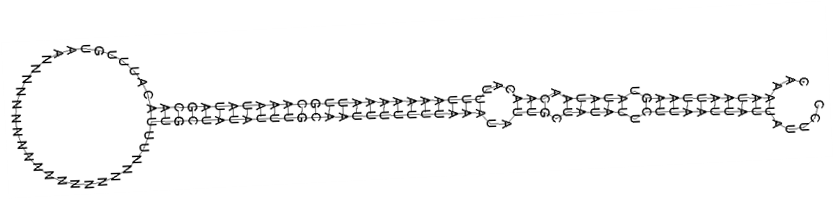


37. miRNA name: PC-3p-99883, got it by deep sequencing and no experiment validated.


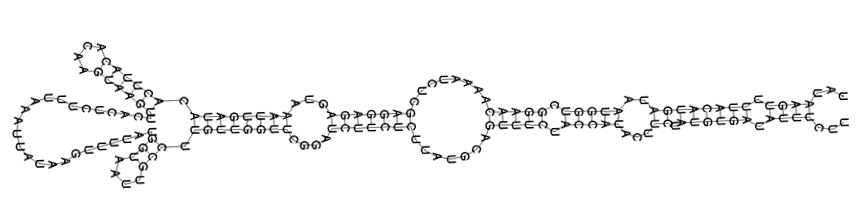


38. miRNA name: PC-3p-104586, got it by deep sequencing and no experiment validated.


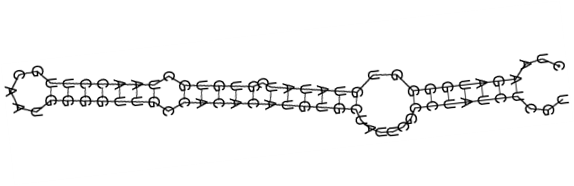


39. miRNA name: PC-3p-112990, got it by deep sequencing and no experiment validated.


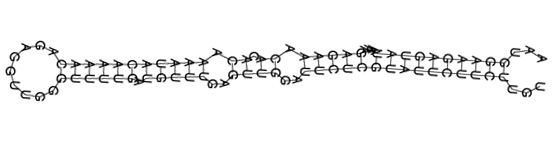


40. miRNA name: PC-3p-139841, got it by deep sequencing and no experiment validated.


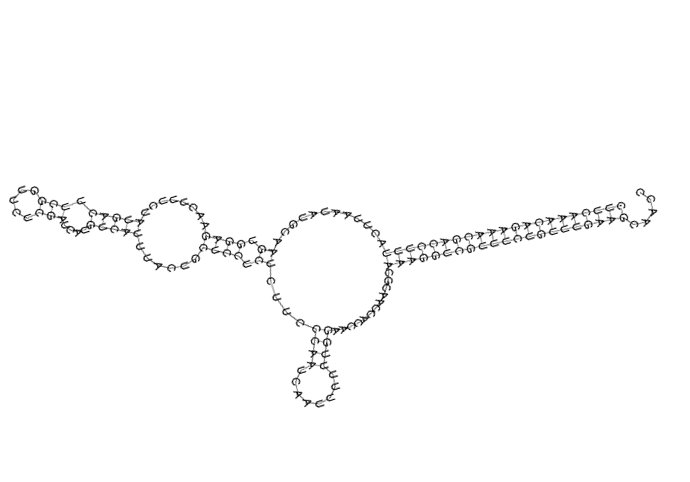


41. miRNA name: PC-3p-148722, got it by deep sequencing and no experiment validated.


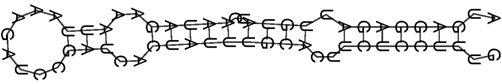


42. miRNA name: PC-3p-149419, got it by deep sequencing and no experiment validated.


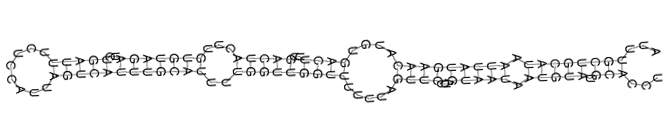


43. miRNA name: PC-3p-157112, got it by deep sequencing and no experiment validated.


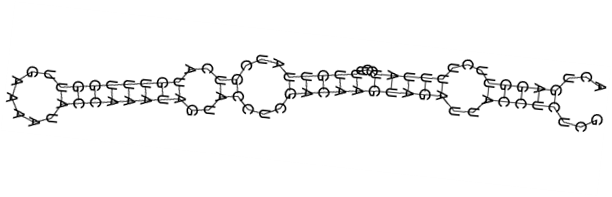


44. miRNA name: PC-3p-157592, got it by deep sequencing and no experiment validated.


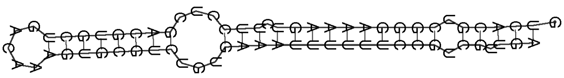


45. miRNA name: PC-3p-158740, got it by deep sequencing and no experiment validated.


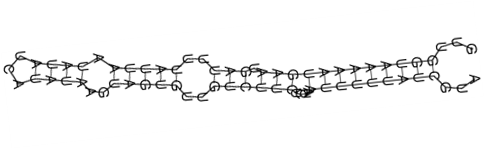


46. miRNA name: PC-3p-167492, got it by deep sequencing and no experiment validated.


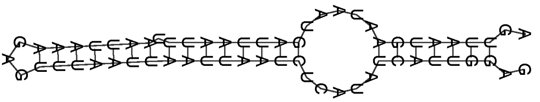


47. miRNA name: PC-3p-175014, got it by deep sequencing and no experiment validated.


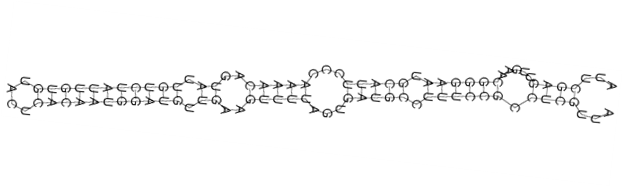


48. miRNA name: PC-3p-202648, got it by deep sequencing and no experiment validated.


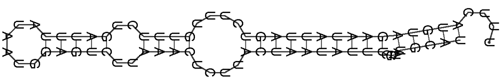


49. miRNA name: PC-3p-209240, got it by deep sequencing and no experiment validated.


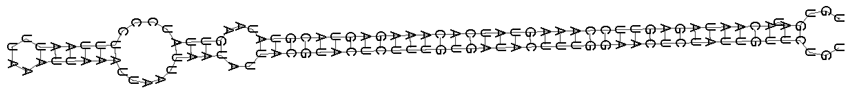


50. miRNA name: PC-3p-209381, got it by deep sequencing and no experiment validated.


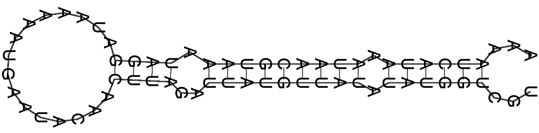


51. miRNA name: PC-3p-222939, got it by deep sequencing and no experiment validated.


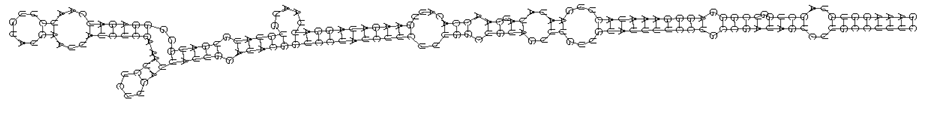


52. miRNA name: PC-3p-223148, got it by deep sequencing and no experiment validated.


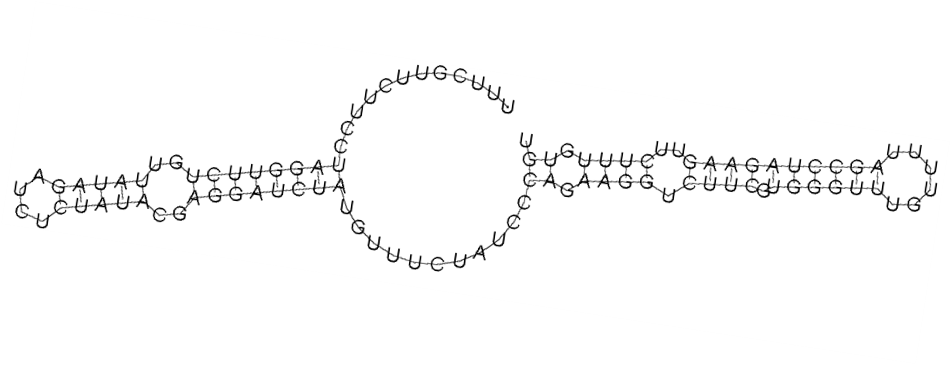


53. miRNA name: PC-3p-225557, got it by deep sequencing and no experiment validated.


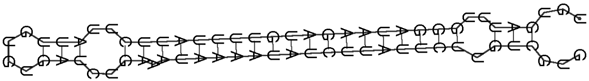


54. miRNA name: PC-3p-232337, got it by deep sequencing and no experiment validated.


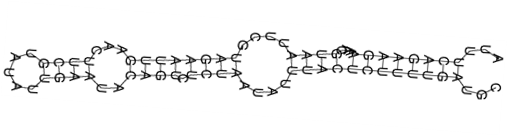


55. miRNA name: PC-3p-254427, got it by deep sequencing and no experiment validated.


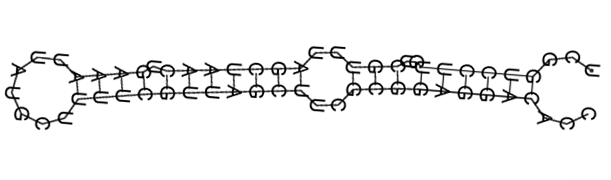


56. miRNA name: PC-3p-271839, got it by deep sequencing and no experiment validated.


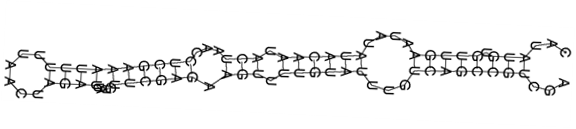


57. miRNA name: PC-3p-297512, got it by deep sequencing and no experiment validated.


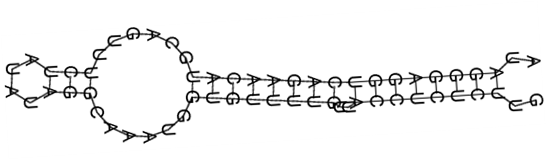


58. miRNA name: PC-3p-298969, got it by deep sequencing and no experiment validated.


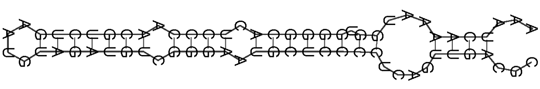


59. miRNA name: PC-3p-311789, got it by deep sequencing and no experiment validated.


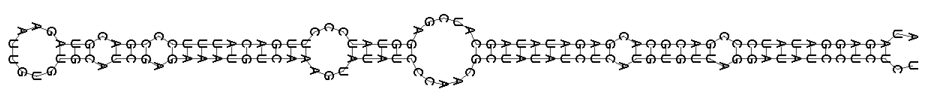


60. miRNA name: PC-3p-318270, got it by deep sequencing and no experiment validated.


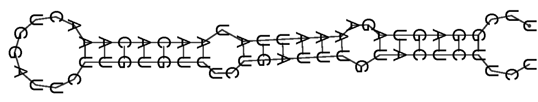


61. miRNA name: PC-3p-318493, got it by deep sequencing and no experiment validated.


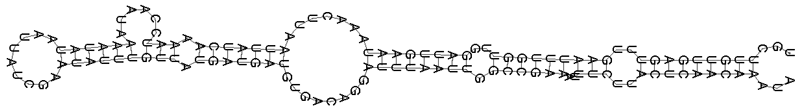


62. miRNA name: PC-3p-322544, got it by deep sequencing and no experiment validated.


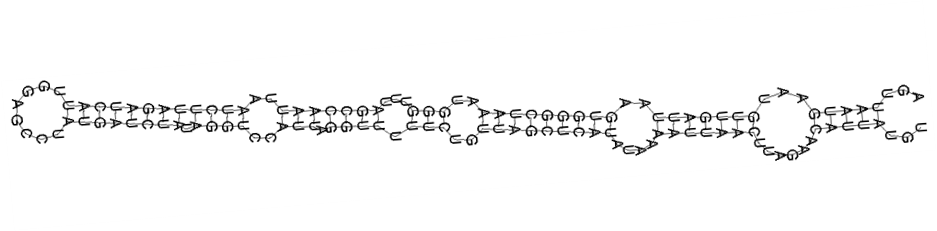


63. miRNA name: PC-5p-7450, got it by deep sequencing and no experiment validated.


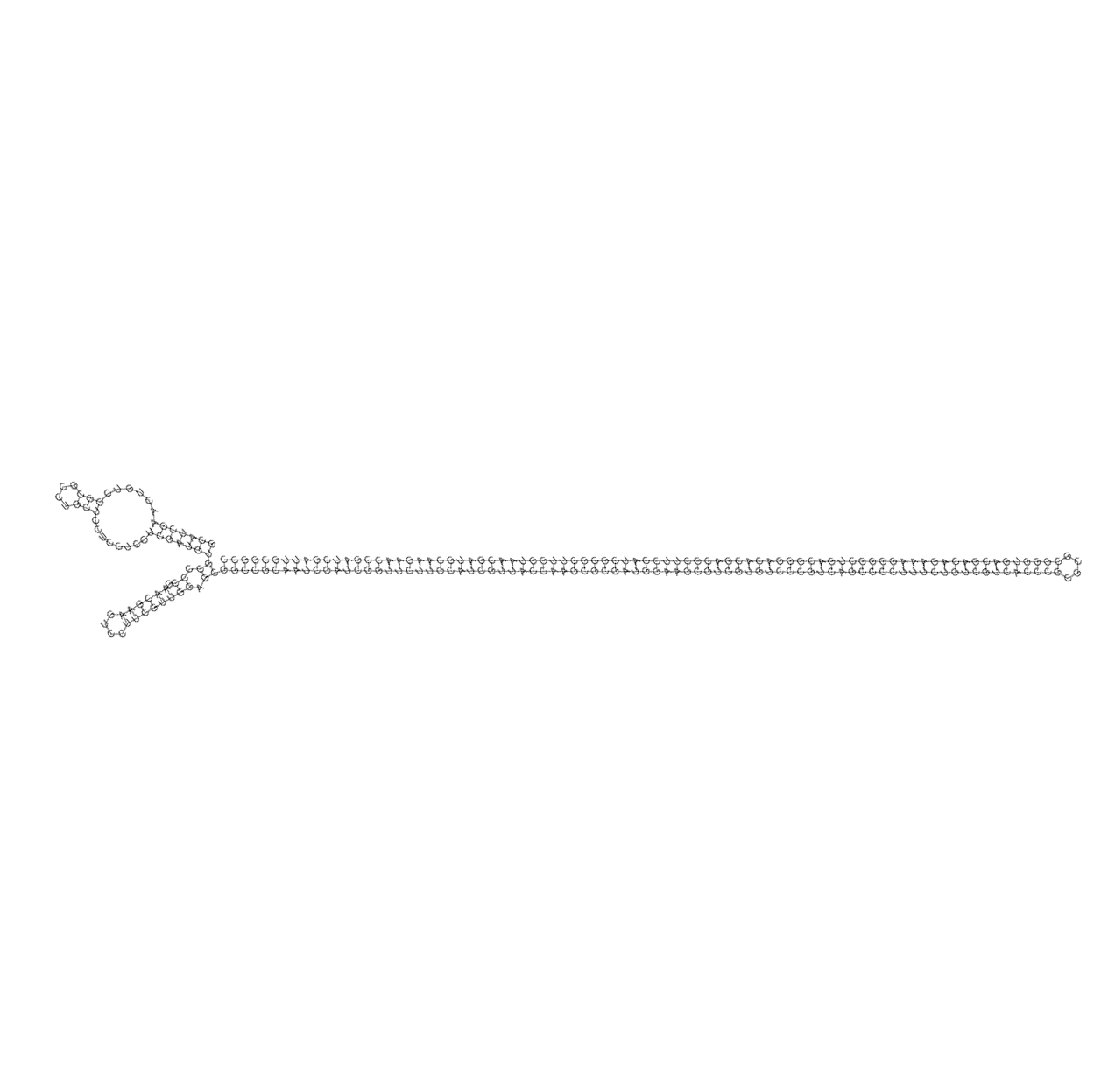


64. miRNA name: PC-5p-9180, got it by deep sequencing and no experiment validated.


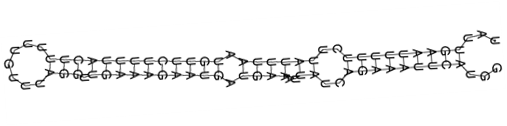


65. miRNA name: PC-5p-17007, got it by deep sequencing and no experiment validated.


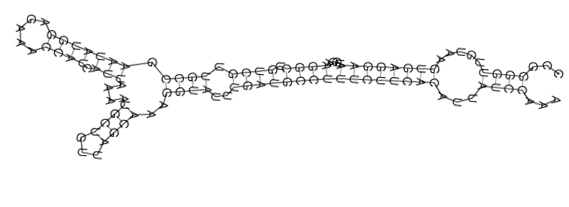


66. miRNA name: PC-5p-19461, got it by deep sequencing and no experiment validated.


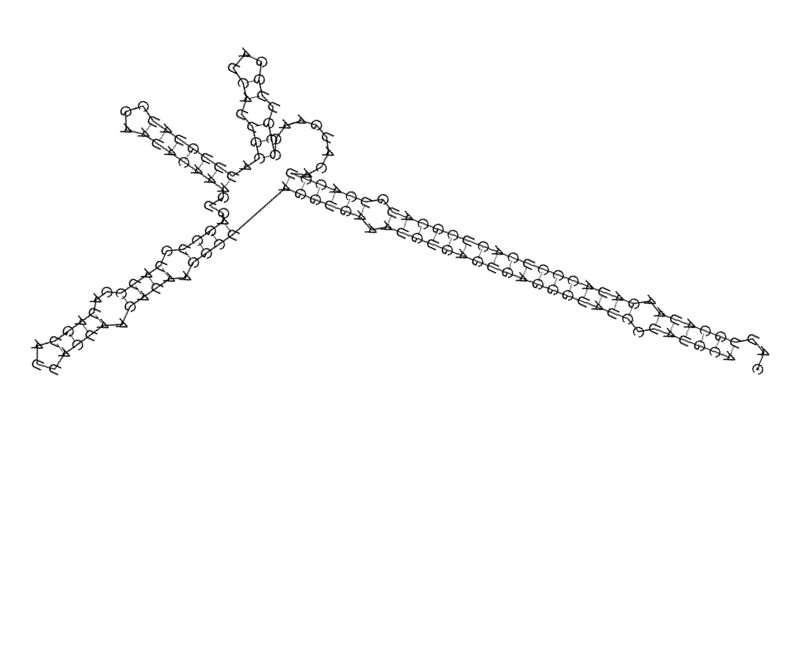


67. miRNA name: PC-5p-21837, got it by deep sequencing and no experiment validated.


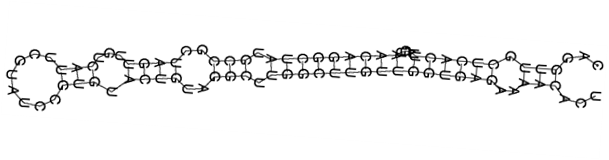


68. miRNA name: PC-5p-22000, got it by deep sequencing and no experiment validated.


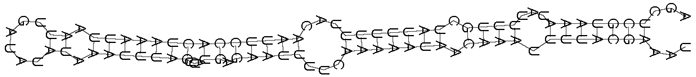


69. miRNA name: PC-5p-23513, got it by deep sequencing and no experiment validated.


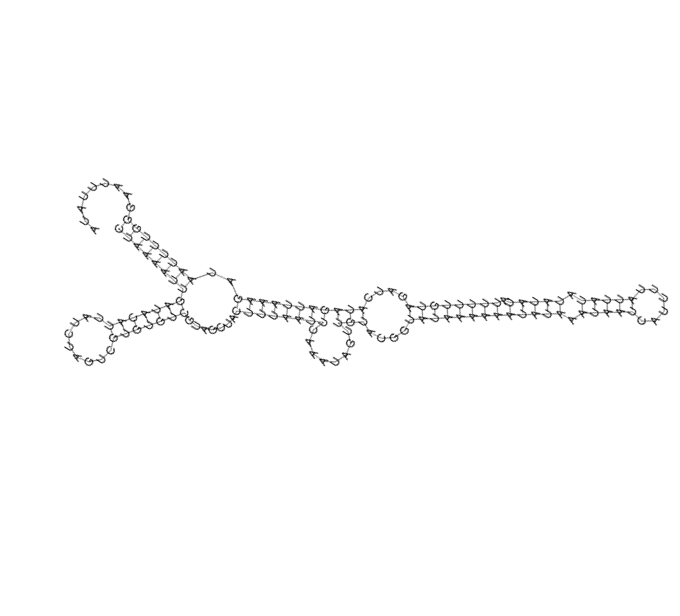


70. miRNA name: PC-5p-23516, got it by deep sequencing and no experiment validated.


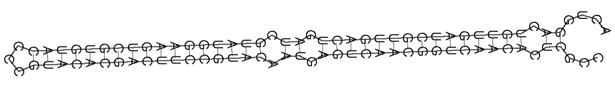


71. miRNA name: PC-5p-24326, got it by deep sequencing and no experiment validated.


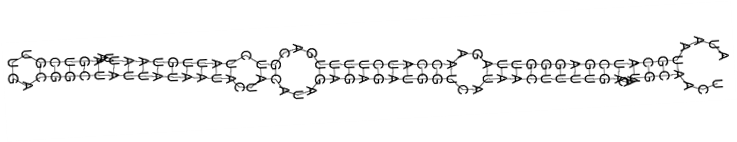


72. miRNA name: PC-5p-26498, got it by deep sequencing and no experiment validated.


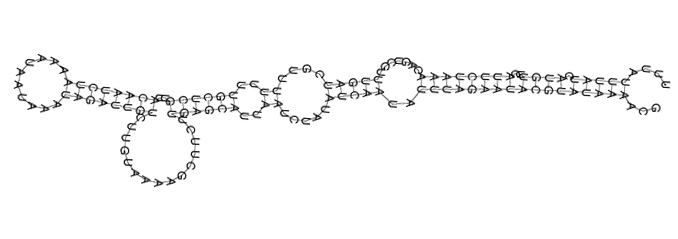


73. miRNA name: PC-5p-28364, got it by deep sequencing and no experiment validated.


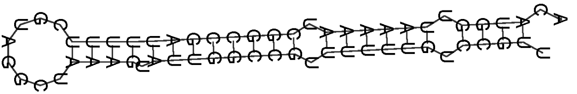


74. miRNA name: PC-5p-34263, got it by deep sequencing and no experiment validated.


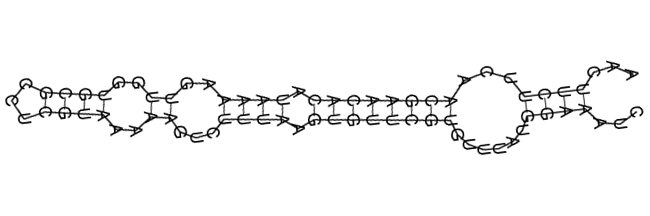


75. miRNA name: PC-5p-36448, got it by deep sequencing and no experiment validated.


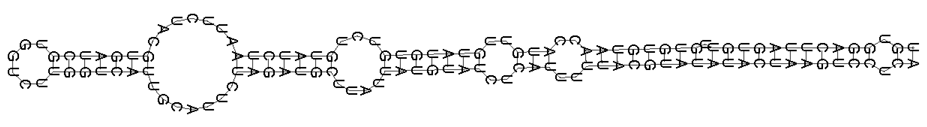


76. miRNA name: PC-5p-38568, got it by deep sequencing and no experiment validated.


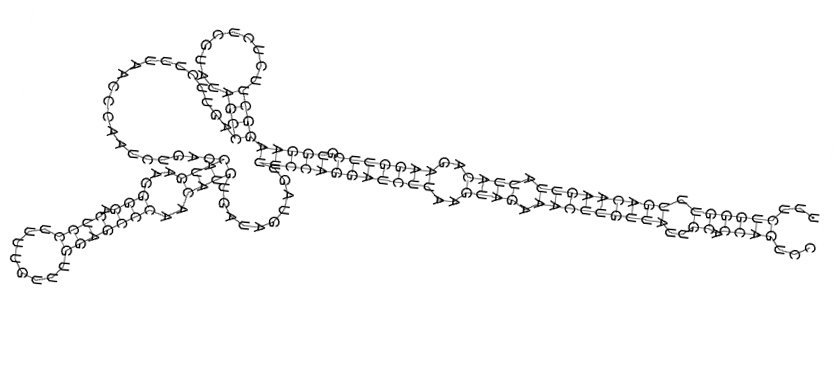


77. miRNA name: PC-5p-46899, got it by deep sequencing and no experiment validated.


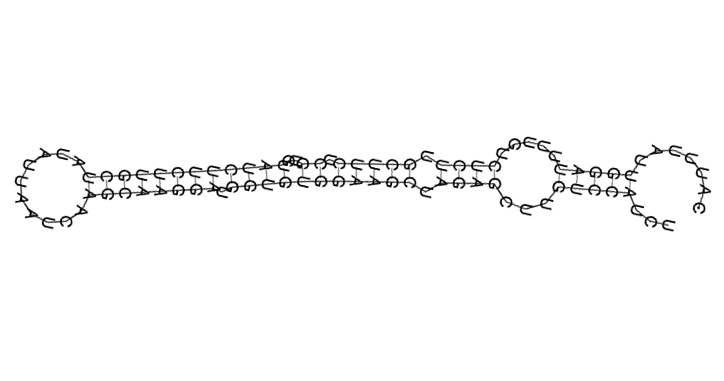


78. miRNA name: PC-5p-51735, got it by deep sequencing and no experiment validated.


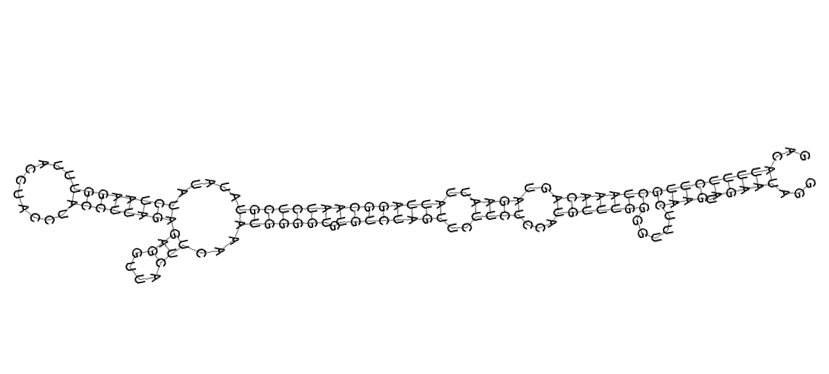


79. miRNA name: PC-5p-53447, got it by deep sequencing and no experiment validated.


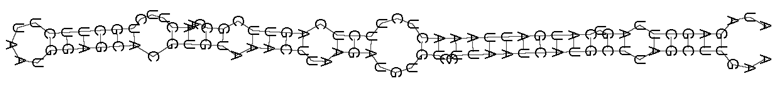


80. miRNA name: PC-5p-53485, got it by deep sequencing and no experiment validated.


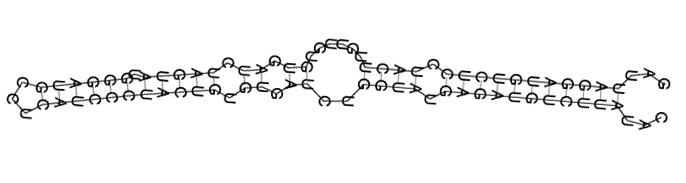


81. miRNA name: PC-5p-58981, got it by deep sequencing and no experiment validated.


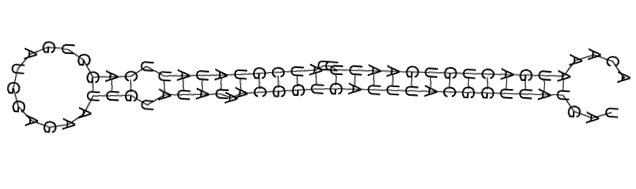


82. miRNA name: PC-5p-61552, got it by deep sequencing and no experiment validated.


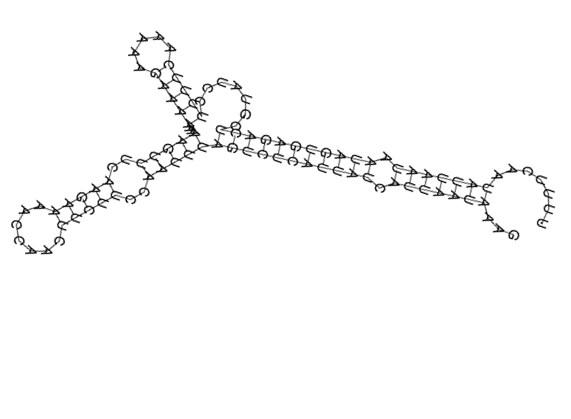


83. miRNA name: PC-5p-61729, got it by deep sequencing and no experiment validated.


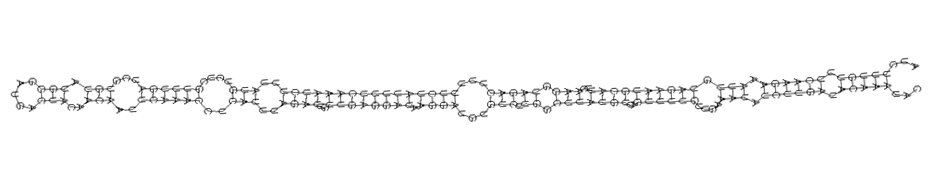


84. miRNA name: PC-5p-63871, got it by deep sequencing and no experiment validated.


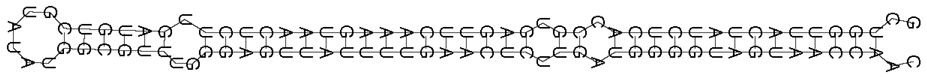


85. miRNA name: PC-5p-65578, got it by deep sequencing and no experiment validated.


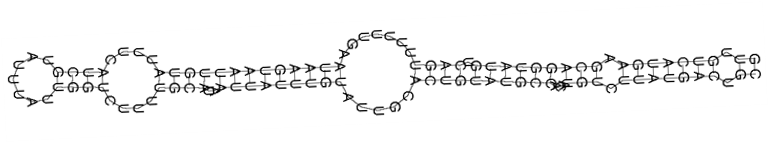


86. miRNA name: PC-5p-66456, got it by deep sequencing and no experiment validated.


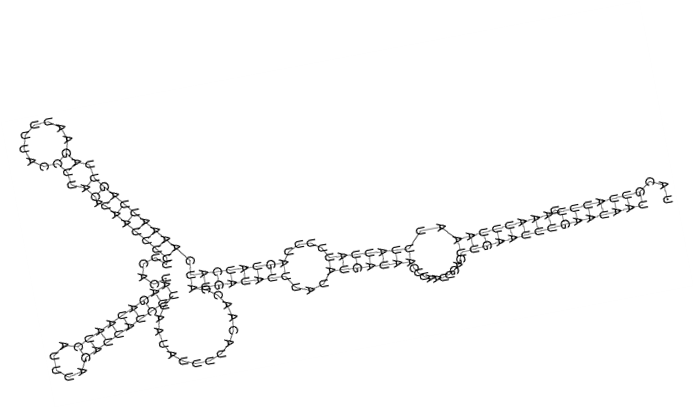


87. miRNA name: PC-5p-66855, got it by deep sequencing and no experiment validated.


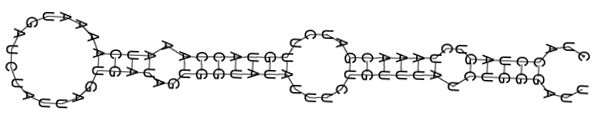


88. miRNA name: PC-5p-67111, got it by deep sequencing and no experiment validated.


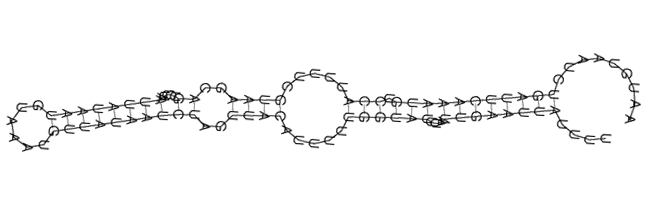


89. miRNA name: PC-5p-67364, got it by deep sequencing and no experiment validated.


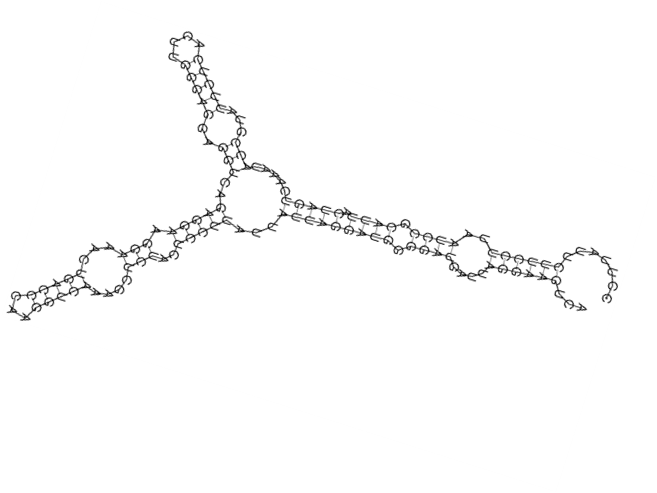


90. miRNA name: PC-5p-67839, got it by deep sequencing and no experiment validated.


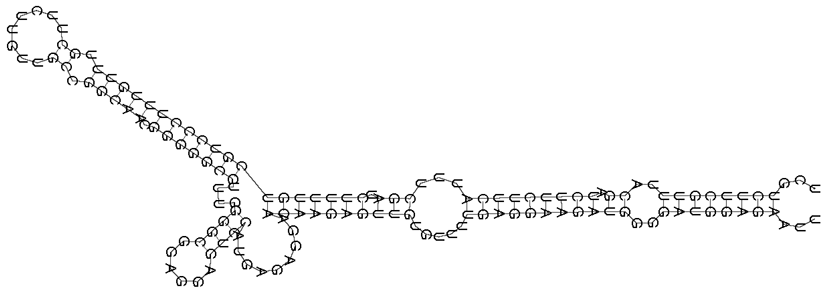


91. miRNA name: PC-5p-70653, got it by deep sequencing and no experiment validated.


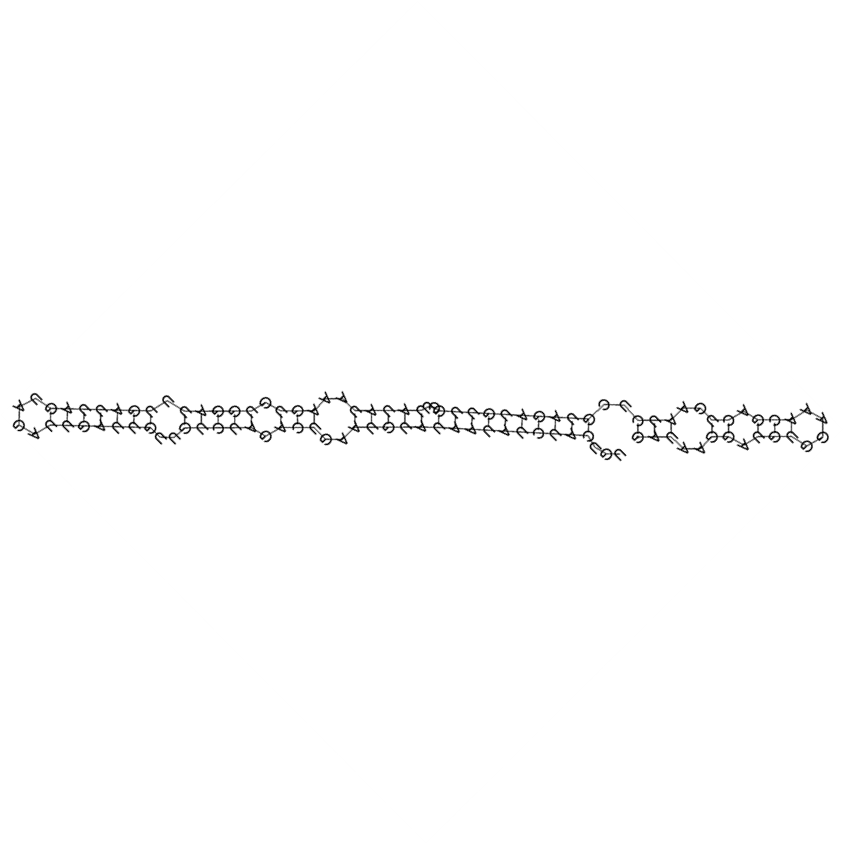


92. miRNA name: PC-5p-72321, got it by deep sequencing and no experiment validated.


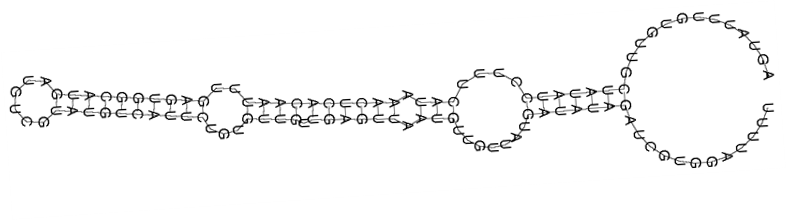


93. miRNA name: PC-5p-7340, got it by deep sequencing and no experiment validated.

94. miRNA name: PC-5p-74136, got it by deep sequencing and no experiment validated.

95. miRNA name: PC-5p-74546, got it by deep sequencing and no experiment validated.

96. miRNA name: PC-5p-76647, got it by deep sequencing and no experiment validated.

97. miRNA name: PC-5p-82400, got it by deep sequencing and no experiment validated.

98. miRNA name: PC-5p-82896, got it by deep sequencing and no experiment validated.

99. miRNA name: PC-5p-84960, got it by deep sequencing and no experiment validated.

100. miRNA name: PC-5p-93097, got it by deep sequencing and no experiment validated.

101. miRNA name: PC-5p-98738, got it by deep sequencing and no experiment validated.

102. miRNA name: PC-5p-100383, got it by deep sequencing and no experiment validated.

103. miRNA name: PC-5p-103503, got it by deep sequencing and no experiment validated.

104. miRNA name: PC-5p-112236, got it by deep sequencing and no experiment validated.

105. miRNA name: PC-3p-25396, got it by deep sequencing and no experiment validated.

106. miRNA name: PC-5p-143739, got it by deep sequencing and no experiment validated.

107. miRNA name: PC-5p-162570, got it by deep sequencing and no experiment validated.

108. miRNA name: PC-5p-172177, got it by deep sequencing and no experiment validated.

109. miRNA name: PC-5p-173172, got it by deep sequencing and no experiment validated.

110. miRNA name: PC-5p-183816, got it by deep sequencing and no experiment validated.

111. miRNA name: PC-5p-187816, got it by deep sequencing and no experiment validated.

112. miRNA name: PC-5p-226754, got it by deep sequencing and no experiment validated.

113. miRNA name: PC-5p-230498, got it by deep sequencing and no experiment validated.

114. miRNA name: PC-5p-244631, got it by deep sequencing and no experiment validated.

115. miRNA name: PC-5p-249593, got it by deep sequencing and no experiment validated.

116. miRNA name: PC-5p-260857, got it by deep sequencing and experiment validated.

117. miRNA name: PC-5p-265341, got it by deep sequencing and no experiment validated.

118. miRNA name: PC-5p-274347, got it by deep sequencing and no experiment validated.

119. miRNA name: PC-5p-322544, got it by deep sequencing and no experiment validated.

120. miRNA name: PC-5p-327097, got it by deep sequencing and no experiment validated.
