# Supplementary material for: High-Throughput Sequencing Identifies Novel and Conserved Cucumber (Cucumis sativus L.) microRNAs in Response to Cucumber Green Mottle Mosaic Virus Infection
Source: PLoS One. 2015 Jun 15;10(6):e0129002. doi: 10.1371/journal.pone.0129002 (PMC4468104; doi:10.1371/journal.pone.0129002)
Supplement: S1 Table — (DOC) [file pone.0129002.s003.doc]

**Table S2**. Predicted candidate (PC) miRNAs isolated by high throughput screening that can be mapped to the cucumber genome.

| No | Mappable reads | copy | miR_seq | start-end position of cucumber genome |
| --- | --- | --- | --- | --- |
| 1 | PC-3p-3557 | 32 | CAAAATGTTGAATTGAA | 4593523-4593781 |
| 2 | PC-3p-4681 | 23 | CTAGTTTACTACGAGGTTGATGATC | 19908267-19908531 |
| 3 | PC-3p-8322 | 12 | TAAAGACTTTTTGCCGATGT | 284804-285063 |
| 4 | PC-3p-20844 | 4 | TTGGTTTTACTTTGTCTCAGTT | 6003408-6003669 |
| 5 | PC-3p-25396 | 4 | TGGATGTCGTCCCTAAGGTAGCC | 17599542-17599804 |
| 6 | PC-3p-28211 | 3 | CATCAAAGTGGTTTCTGGTAAC | 2455570-2455831 |
| 7 | PC-3p-31277 | 3 | ATCTATGTTGGAATCAATGTTG | 2751606-2751867 |
| 8 | PC-3p-34277 | 3 | CTGCATTATTGCTCTTTGATTCT | 30218574-30218836 |
| 9 | PC-3p-37117 | 3 | AATTTGATCTTCTGATTCTCTC | 14400613-14400874 |
| 10 | PC-3p-37565 | 3 | CTTCCTCTCACAGTTTATCTCC | 12630819-12631080 |
| 11 | PC-3p-38361 | 3 | CTATGCTAGCGTTATTAAAGGCC | 14249789-14250051 |
| 12 | PC-3p-39976 | 2 | AACAATTTGTACCGTACTTC | 15421502-15421761 |
| 13 | PC-3p-41177 | 2 | CTTCGATCTCATCTCACTTCTTCCA | 14299561-14299825 |
| 14 | PC-5p-44253 | 2 | AGTTACATCTAGTGGTTACTAAT | 7944669-7944931 |
| 15 | PC-3p-45338 | 2 | TTTGATCGATTTATCCAAAACAC | 10904023-10904285 |
| 16 | PC-3p-46648 | 2 | GGTTTTGAATTTTTTATTGAAT | 10620580-10620841 |
| 17 | PC-3p-47140 | 2 | CTTATCTTGAGCGCACACATG | 19031985-19032245 |
| 18 | PC-3p-48988 | 2 | ATTGGACAGAGAAGTATATC | 16922159-16922418 |
| 19 | PC-3p-50340 | 2 | GGGGTTCTATTCTACTATTC | 17596065-17596324 |
| 20 | PC-3p-50452 | 2 | TTTCATTCGTGAGCTTTTACA | 12039243-12039503 |
| 21 | PC-3p-50621 | 2 | ATGACTGGAAAATAATTGTCG | 6501731-6501991 |
| 22 | PC-3p-50720 | 2 | AGTTGATCTCGAACATTGATCC | 8296776-8297037 |
| 23 | PC-3p-51738 | 2 | TATGATCTCCTTATTTCTGGTC | 6945453-6945714 |
| 24 | PC-3p-59087 | 2 | TTGACAAGTTTATCACTTCCATA | 8517998-8518260 |
| 25 | PC-3p-59313 | 2 | ATTTTATCTCGCAAACATC | 20322030-20322288 |
| 26 | PC-3p-67700 | 2 | TTTGTTTTACTTTGTCTTAGTT | 3504295-3504556 |
| 27 | PC-3p-69764 | 2 | CTTGTCTGTTAGTGAATCTTCC | 7892868-7893129 |
| 28 | PC-3p-70101 | 2 | TTTATATTTGTTTCATTATTCCATG | 2608387-2608651 |
| 29 | PC-3p-70771 | 2 | ATTTGTCCTTGTAAAATACTGTC | 21202170-21202432 |
| 30 | PC-3p-71762 | 2 | CATATTCTATAATTGTATGATATC | 30367577-30367840 |
| 31 | PC-5p-72026 | 2 | TCCTCAAATTCTTTGAGCC | 1-140 |
| 32 | PC-3p-76546 | 1 | TTTTCTAACCCAACTCAA | 945668-945925 |
| 33 | PC-3p-87233 | 1 | GTTTAGTCTTCACCATGT | 9765547-9765804 |
| 34 | PC-3p-87931 | 1 | TTCTAAATTTTTGTCAATGTAC | 832131-832392 |
| 35 | PC-3p-88917 | 2 | CTTTGTCGTGTGCTAACC | 4600799-4601056 |
| 36 | PC-3p-95926 | 1 | TTGCTATATTCTTAATTATTATTCC | 8486318-8486582 |
| 37 | PC-3p-99883 | 2 | CTTGTTGGTTCGGATCTTC | 18020502-18020760 |
| 38 | PC-3p-104586 | 1 | CCACACATGTGCTATTCGCCTATC | 16594-16857 |
| 39 | PC-3p-112990 | 1 | GTTTTGATGTTTGAGTTGGATTC | 18714225-18714487 |
| 40 | PC-3p-139841 | 1 | AAGGTCGTTTCTGTTTGAAGCAACC | 17139233-17139497 |
| 41 | PC-3p-148722 | 2 | TCCGATCACTATTTGCACTTC | 1582362-1582622 |
| 42 | PC-3p-149419 | 1 | TTAGTTCTTAGTAATAATG | 9464602-9464860 |
| 43 | PC-3p-157112 | 1 | GTACCTCGACAAGTAGATTACCTC | 12291571-12291834 |
| 44 | PC-3p-157592 | 1 | GTGCGTCTGTGAAATTTTTTCCG | 14894624-14894886 |
| 45 | PC-3p-158740 | 1 | TAGTGTTGTCTTCTCTAATTTTTA | 5321983-5322246 |
| 46 | PC-3p-167492 | 2 | TTTAGTTAATTAATCTCATA | 17352318-17352577 |
| 47 | PC-3p-175014 | 1 | GTTTTAGTGATGCCTTTCCGCC | 7404838-7405099 |
| 48 | PC-3p-202648 | 2 | GTCTTAAACTCTTACTAATATT | 8273983-8274244 |
| 49 | PC-3p-209240 | 1 | TTTGTGATACTTTGGAACTCTA | 17333796-17334057 |
| 50 | PC-3p-209381 | 1 | ACTTAGATTATGTTATATATGG | 16995429-16995690 |
| 51 | PC-3p-222939 | 1 | TGATTATTGGATACGGTCCTATCT | 18187958-18188221 |
| 52 | PC-3p-223148 | 1 | GTTTGTTTTAGCCTATAAGTTCT | 11153113-11153375 |
| 53 | PC-3p-225557 | 1 | CTGAAATAAAATATCTTATTCTTG | 13133770-13134033 |
| 54 | PC-3p-232337 | 1 | TACAATCTCTAATATTTAC | 1-203 |
| 55 | PC-3p-254427 | 1 | TTTCGTTAGCTTCGCGGAGGACACC | 5707220-5707484 |
| 56 | PC-3p-271839 | 1 | ATTATGTTCGAGAAGTTTTGTAT | 14718-14980 |
| 57 | PC-3p-297512 | 1 | AAATGGTGTTTTGTTACCTCTC | 1572644-1572905 |
| 58 | PC-3p-298969 | 1 | ATGTAGATGTCGGGAATGCTCCCC | 13340890-13341153 |
| 59 | PC-3p-311789 | 1 | TATATCTCATGTGTTAGGATATCC | 5744327-5744590 |
| 60 | PC-3p-318270 | 1 | GTTTCTGATTTGTACTCTTCT | 37772923-37773183 |
| 61 | PC-3p-318493 | 1 | ATTTAATTGGCCGAAATTTCTTAC | 16915070-16915333 |
| 62 | PC-5p-322544 | 1 | TTTAGCCAATTAATCTTAGA | 15096939-15097198 |
| 63 | PC-5p-7450 | 18 | GTTACCAAGCGCGATGGAAGCG | 13598085-13598346 |
| 64 | PC-5p-9180 | 10 | CTTATTTAATGTTCTTT | 6983905-6987162 |
| 65 | PC-5p-17007 | 7 | AAGTGAGGAATGCAAGGCTG | 3749439-3749698 |
| 66 | PC-5p-19461 | 5 | CATTGCATAAGATACCCTCAC | 13020951-13021211 |
| 67 | PC-5p-21837 | 5 | CTATGCCGCTAGTTGTCAT | 35054512-35054770 |
| 68 | PC-5p-22000 | 4 | CTATTTTTTACAATTCC | 16013256-16013512 |
| 69 | PC-5p-23513 | 4 | CTAAAATTGATACATTATCTAG | 24524395-24524656 |
| 70 | PC-5p-23516 | 4 | CTGGACTGTTGATCGTTGACTGA | 16070751-16071013 |
| 71 | PC-5p-24326 | 5 | AATGCATCGAGGGTTAGAACCA | 19348572-19348833 |
| 72 | PC-5p-26498 | 3 | TATCATGTTCATTCTAAACAG | 13872500-13872760 |
| 73 | PC-5p-28364 | 3 | GTTAAAAATCGGCCGATTTT | 19225834-19226039 |
| 74 | PC-5p-34263 | 3 | AACACATAAAAGTTGGTGCGCC | 31352460-31352721 |
| 75 | PC-5p-36448 | 3 | CTTAGTGTTGTGTGTAACCATG | 2529997-2530528 |
| 76 | PC-5p-38568 | 3 | CAGAAGGTTCGTGGAAGGCTTCTC | 1321848-1322111 |
| 77 | PC-5p-46899 | 2 | ATTGGATTTTGTCTCTTGCTTC | 6243739-6244000 |
| 78 | PC-5p-51735 | 2 | GTAGAATTATTAGGCAATC | 25558522-25558780 |
| 79 | PC-5p-53447 | 2 | GAGCTTAGTCATGATTAAACTCTTC | 14405935-14406199 |
| 80 | PC-5p-53485 | 2 | GTGTGATCTAGTATGGGATG | 17132979-17133238 |
| 81 | PC-5p-58981 | 2 | ACAAATGACTGTGAATTTTA | 16211377-16211636 |
| 82 | PC-5p-61552 | 2 | TCAATATTAATAATAGTGAGAC | 6136777-6137038 |
| 83 | PC-5p-61729 | 2 | CTATTTTCAAATCTTTATGTC | 8-203 |
| 84 | PC-5p-63871 | 2 | CGTGAGTTCAAAGTAACTCTTGATG | 8535476-8535740 |
| 85 | PC-5p-65578 | 2 | GAATAAGTAATTGTATTTCA | 1726382-1726641 |
| 86 | PC-5p-66456 | 2 | ATTATTTTTAGTATCATC | 17539322-17539579 |
| 87 | PC-5p-66855 | 2 | CTACCTAGTCTAAACGATC | 17141316-17141574 |
| 88 | PC-5p-67111 | 2 | TCTGATTCAAATGTCCATTT | 457210-457469 |
| 89 | PC-5p-67364 | 3 | CTTCCTTAATCCGCATTACTAG | 1-245 |
| 90 | PC-5p-67839 | 2 | GTTTACGATCTTCTTCATTTCGA | 27986046-27986308 |
| 91 | PC-5p-70653 | 2 | ATAAGGATGTGGAAACGACTGAATC | 15866521-15866789 |
| 92 | PC-5p-72321 | 2 | AATTTGAGTGGCATGATG | 17115780-17116037 |
| 93 | PC-5p-73408 | 2 | GGAATCTTCCCAATATATTGACTA | 15533938-15534201 |
| 94 | PC-5p-74136 | 2 | ATAGACTTGTATCACTAATAGAC | 19157132-19157394 |
| 95 | PC-5p-74546 | 1 | CTTGTATCACTGATAAAC | 11244345-11244602 |
| 96 | PC-5p-76647 | 2 | ACTCAGACTGTCCTACATTCAACTA | 9005992-9006256 |
| 97 | PC-5p-82400 | 1 | CATGTATGATTTAATTACTAATC | 1077268-1077530 |
| 98 | PC-5p-82896 | 1 | GTTTTTGTTTTCGCCCCAAAACGGG | 10486835-10487099 |
| 99 | PC-5p-84960 | 1 | ATTTATATTAATAATAGTGAGAC | 25154330-25154592 |
| 100 | PC-5p-93097 | 1 | ACTCCTTTTTATAATTATTATC | 20736606-20736867 |
| 101 | PC-5p-98738 | 1 | ATTTATGAGGTAGATAACCAACCA | 14953500-14953763 |
| 102 | PC-5p-100383 | 1 | CAAAACTATTGCTGATAGAC | 10794048-10794307 |
| 103 | PC-5p-103503 | 2 | ATAAGGTTTGTGGAAGGTTTCTC | 14402343-14402605 |
| 104 | PC-5p-112236 | 1 | CGTGAGATTTCTATAATATGCCTGC | 8923137-8923401 |
| 105 | PC-5p-135464 | 1 | AGACTTGTATCGATGATACACT | 28993722-28993983 |
| 106 | PC-3p-143739 | 1 | CTTCGCGGTAAGTCTAACCCTAATT | 10966557-10966821 |
| 107 | PC-5p-162570 | 1 | CACATAAGATTATCCTTAGATCT | 16160500-16160762 |
| 108 | PC-5p-172177 | 1 | TGATCTATATATGTTTCTGAGG | 7475579-7475840 |
| 109 | PC-5p-173172 | 2 | ATTTAGGATATATACTCAC | 20492920-20493178 |
| 110 | PC-5p-183816 | 1 | CTTGTATCGCTGATAGATT | 10362240-10362498 |
| 111 | PC-5p-187816 | 1 | AAGCCTAATATTGCAATTCTCTGA | 22885626-22885889 |
| 112 | PC-5p-226754 | 1 | TTAATATTATATTTTCTCTATTT | 13815138-13815400 |
| 113 | PC-5p-230498 | 1 | AAACTATCACTGATAAAC | 15890263-15890520 |
| 114 | PC-5p-244631 | 1 | GTGGGCTTGGCCTCATTTGC | 5593259-5593518 |
| 115 | PC-5p-249593 | 1 | ATAGACTTGTATCACTCATAGAC | 16736397-16736659 |
| 116 | PC-5p-260857 | 1 | ATGAACGACAGCGACTTCC | 4022816-4023074 |
| 117 | PC-5p-265341 | 1 | TAGTCATAGTATCGCATG | 2637337-2637594 |
| 118 | PC-5p-274347 | 1 | ATAGCAAAACTGTCGCTGATAGAC | 16958521-16958784 |
| 119 | PC-5p-322544 | 1 | TTTAGCCAATTAATCTTAGA | 16547653-16547912 |
| 120 | PC-5p-327097 | 1 | ACAAATATATCCAAATCCA | 3666387-3666645 |
