# Supplementary material for: High-Throughput Sequencing Identifies Novel and Conserved Cucumber (Cucumis sativus L.) microRNAs in Response to Cucumber Green Mottle Mosaic Virus Infection
Source: PLoS One. 2015 Jun 15;10(6):e0129002. doi: 10.1371/journal.pone.0129002 (PMC4468104; doi:10.1371/journal.pone.0129002)
Supplement: S2 Table — (DOCX) [file pone.0129002.s004.docx]

Table S1. Novel cucumber miRNAs identified by high-throughput sequencing.

| miRNA name | Sequence (5'-3') | L (nt) | Copy | MFE (kcal/ml) | MEFI | Match site | Gene name | Target description |
| --- | --- | --- | --- | --- | --- | --- | --- | --- |
| csa-miRn1-3p | CTCGCAAACATCTGGTAGACATC | 23 | 29 | -19.50 | 0.9 | 9123605-9123867 | Csa1M109320.1 | regulation of gene expression,  response to abiotic and biotic stress,  signaling pathway,  transcription,  seed coat development,  DNA/protein binding,  nucleus component. |
|  |  |  |  |  |  |  | Csa6M505970.1 | uncharacterized |
|  |  |  |  |  |  |  | Csa7M073450.1 | UDP-glycosyltransferase 73B3-like, scopoletin glucosyltransferase-like |
|  |  |  |  |  |  |  | Csa3M854260.1 | putative pentatricopeptide repeat-containing protein At1g19290-like |
| csa-miRn2-3p | CGTTGAGTATTATCTTTCAGTTC | 23 | 13 | -35.40 | 0.8 | 4354896-4355158 | Csa6M056520.1 | mediated signaling pathway,  response to abiotic and biotic stress,  regulation of transcription, flower development and gene specific transcription.  protein folding,  seed germination,  protein binding,  membrane, nucleus component. |
|  |  |  |  |  |  |  | Csa6M488360.1 | BTB/POZ domain-containing protein At5g66560-like |
| csa-miRn3-3p | CTTGTGGGTAATAGGCTTTCCTTCCTTG | 28 | 4 | -27.80 | 0.7 | 18576407-18576674 | Csa4M578870.1 | ion transport,  cAMP / DNA binding,  membrane and extracellular region component. |
|  |  |  |  |  |  |  | Csa5M166410.2 | 60S ribosomal protein L36-2-like |
|  |  |  |  |  |  |  | Csa5M166410.1 | 60S ribosomal protein L36-2-like |
| csa-miRn4-5p | ACTTCGCGGTAAGTCTAACCCTAATT | 26 | 1 | -70.40 | 1 | 14602553-14602818 | unknown | unknown |
| csa-miRn5-5p | AATTACTATAATAACACCTTCACAT | 25 | 2 | -18.90 | 0.7 | 620652-620916 | Csa4M056510.1  Csa2M033380.1 | Pectin acetylesterase, cytoskeleton organization,  actin binding / cytoskeleton |
|  |  |  |  |  |  |  | Csa3M912290.1 | uncharacterized |
|  |  |  |  |  |  |  | Csa1M616240.1 | chromosome |
|  |  |  |  |  |  |  | Csa3M019350.1 | putative dual specificity protein phosphatase DSP8-like |
| csa-miRn6-3p | TCTTCTTCCTCTTCCTTTCT | 20 | 2 | -15.31 | 0.4 | 12155069-12155328 | Csa2M033380.1  Csa7M219220.1  Csa3M683670.1      Csa3M117970.1  Csa2M360000.1 | actin binding / cytoskeleton  unknown  lipid biosynthetic process,  acyltransferase activity,  zinc ion binding,  membrane / thylakoid component  unknown  unknown |
|  |  |  |  |  |  |  | Csa3M019980.1 | zinc finger protein ZAT9-like |
|  |  |  |  |  |  |  | Csa2M380020.2 | serine/arginine repetitive matrix protein 2 |
|  |  |  |  |  |  |  | Csa2M380020.4 | serine/arginine repetitive matrix protein 2 |
|  |  |  |  |  |  |  | Csa2M380020.1 | serine/arginine repetitive matrix protein 2 |
|  |  |  |  |  |  |  | Csa2M380020.3 | uncharacterized |
|  |  |  |  |  |  |  | Csa3M119700.1 | probable WRKY transcription factor 41-like |
|  |  |  |  |  |  |  | Csa4M000700.1 | telomere repeat-binding factor 1-like |
|  |  |  |  |  |  |  | Csa1M424880.1 | pathogenesis-related homeodomain protein-like |
|  |  |  |  |  |  |  | Csa6M079210.1 | uncharacterized |
|  |  |  |  |  |  |  | Csa3M690290.1 | DNA topoisomerase 2-like |
|  |  |  |  |  |  |  | Csa6M104100.1 | pre-mRNA-processing factor 6-like |
|  |  |  |  |  |  |  | Csa1M605660.1 | Putatide/nitrate transporter At5g 19640-like |
|  |  |  |  |  |  |  | Csa3M182050.1 | O-glucosyltransferase rumi homolog |
|  |  |  |  |  |  |  | Csa1M097640.1 | unknown |
|  |  |  |  |  |  |  | Csa7M357030.1 | transcription initiation factor TFIID subunit 1 |
|  |  |  |  |  |  |  | Csa3M232950.1 | probable serine/threonine-protein kinase At1g54610-like |
|  |  |  |  |  |  |  | Csa6M502040.1 | globulin-1 S allele |
|  |  |  |  |  |  |  | Csa5M608280.2 | protein CHUP1, chloroplastic-like |
|  |  |  |  |  |  |  | Csa5M608280.1 | protein CHUP1, chloroplastic-like |
|  |  |  |  |  |  |  | Csa6M361300.1 | chromosome / protein AF-9 |
|  |  |  |  |  |  |  | Csa1M144290.1 | vacuolar protein sorting-associated protein 41 homolog |
|  |  |  |  |  |  |  | Csa2M020850.1 | chromosome |
|  |  |  |  |  |  |  | Csa6M425750.1 | chromosome |
|  |  |  |  |  |  |  | Csa6M425750.2 | chromosome |
|  |  |  |  |  |  |  | Csa6M516920.1 | sugar carrier protein C-like |
|  |  |  |  |  |  |  | Csa1M007860.1 | serine / threonine-protein kinase AtPK2/AtPK19-like |
|  |  |  |  |  |  |  | Csa3M912940.1 | Bax inhibitor 1-like, transcript variant 2 |
|  |  |  |  |  |  |  | Csa1M707120.1 | E3 ubiquitin-protein ligase BOI-like |
|  |  |  |  |  |  |  | Csa6M525380.1 | FACT complex subunit SPT16-like |
|  |  |  |  |  |  |  | Csa3M777630.1 | putative protein TPRXL |
| csa-miRn7-5p | AGGTGGTTGGAGGTTGGTTCA | 21 | 7 | -47.20 | 0.8 | 310605-310865 | unknown | unknown |
| csa-miRn8-3p | GTTGGGAGGATGGAGCGGTT | 20 | 2 | -23.20 | 0.6 | 18772506-18772765 | Csa3M849900.1 | glutamate receptor 2.7-like |
|  |  |  |  |  |  |  | Csa7M432440.1 | pre-mRNA-processing-splicing factor 8-like |
|  |  |  |  |  |  |  | Csa3M806260.1 | probable L-type lectin-domain containing receptor kinase S.5-like |
|  |  |  |  |  |  |  | Csa7M257340.1 | polyol transporter 5-like |
|  |  |  |  |  |  |  | Csa5M161290.1 | nitrate transporter 1.1-like |
