# Supplementary material for: High-Throughput Sequencing Identifies Novel and Conserved Cucumber (Cucumis sativus L.) microRNAs in Response to Cucumber Green Mottle Mosaic Virus Infection
Source: PLoS One. 2015 Jun 15;10(6):e0129002. doi: 10.1371/journal.pone.0129002 (PMC4468104; doi:10.1371/journal.pone.0129002)
Supplement: S3 Table — (DOC) [file pone.0129002.s005.doc]

**Table S3.** Expression of 88 miRNAs in different organs and at different time points post inoculation with CGMMV.

| **transcript name** | **10 dpi*** | **30 dpi_2_*** | **50 dpi_3_*** | **mfi*** | ffi***** |
| --- | --- | --- | --- | --- | --- |
| bdi-MIR390-p3 | -*0.65* | -*0.67* | *0.14* | *0.55* | -*0.14* |
| sbi-MIR159b-p3 | -*0.36* | -*0.96* | *0.26* | *0.52* | -*0.37* |
| aly-miR169n | ***1.20*** | *0.93* | *-0.07* | *0.21* | *0.07* |
| aly-miR164a | **-1.04** | *0.36* | *0.01* | *-0.36* | *-0.36* |
| cme-miR164 | **-1.09** | *0.37* | *0.04* | *-0.23* | *0.21* |
| tcc-miR164c | **-0.90** | ***0.15*** | ***-0.01*** | *-0.42* | *0.10* |
| aly-miR172e | ***1.47*** | *0.19* | *-0.07* | *-0.09* | *-0.61* |
| aly-miR172c | *1.47* | *0.23* | *-0.14* | *-0.23* | *-0.61* |
| aly-miR172c stdev | ***2.45*** | *4.71* | # | # | # |
| tcc-miR172a | ***1.33*** | *0.28* | *-0.12* | *-0.26* | *-0.52* |
| ath-miR172e | ***1.41*** | *0.21* | *-0.06* | *-0.14* | *-0.25* |
| aly-miR172a | ***1.43*** | *0.16* | *-0.1* | *-0.09* | *-0.7* |
| aly-miR399b | **1.07** | *-0.82* | *0.97* | *0.15* | *0.80* |
| rco-miR167a | *0.21* | *-0.25* | *0.08* | *-0.10* | *-0.03* |
| aly-miR838 | *-0.42* | ***-2.07*** | *0.29* | *0.39* | *-0.13* |
| **csa-miRn6-3p** | *-0.77* | ***-1.74*** | *0.08* | *0.61* | *-0.02* |
| ath-miR5021 | *-0.34* | **-1.88** | *0.27* | *0.40* | *-0.06* |
| ath-miR854a | *-0.06* | **-1.81** | *-0.05* | *0.38* | *-0.26* |
| **csa-miRn8-3p** | *-0.65* | ***-1.93*** | *-0.15* | *0.42* | *0.01* |
| mtr-MIR2673a-p5 | *-0.61* | ***-1.51*** | *0.18* | *0.67* | *-0.15* |
| peu-miR2916 | *0.34* | **-0.79** | *0.53* | *0.23* | *-0.44* |
| ath-miR5020c | *-0.40* | ***-1.46*** | *0.51* | *0.32* | *-0.29* |
| aly-miR853 | *0.92* | **-1.39** | *0.02* | *0.78* | *-0.32* |
| aly-miR834 | *0.02* | **-1.53** | *0.57* | *0.24* | *-0.29* |
| aly-miR4249 | # | ***-1.95*** | # | # | # |
| tcc-miR319 | *0.01* | *-0.11* | ***-0.86*** | *-0.31* | *0.08* |
| ptc-miR319i | # | # | *-1.21* | *-0.21* | *-0.35* |
| ptc-miR319a | *0.38* | *-0.53* | **-0.92** | **-0.68** | *0.16* |
| ptc-miR319e | *0.45* | *0.26* | **-0.81** | **-0.68** | *0.15* |
| aly-miR156a | *-0.18* | *0.47* | **-1.01** | *-0.43* | *0.31* |
| gma-MIR169g-p5 | *-0.21* | *-2.22* | *0.43* | **0.60** | *-0.21* |
| aly-miR394a | # | *1.72* | # | # | *-0.03* |
| ptc-miR397b | # | # | # | *-0.54* | *0.27* |
| aly-miR159b | *0.47* | *-0.05* | *-0.49* | *-0.62* | *0.09* |
| aly-miR164b | *0.53* | *4.06* | *3.63* | # | # |
| tcc-miR169i | *-1.14* | *-0.41* | *0.51* | # | # |
| tcc-miR530a | *0.59* | *0.77* | *0.08* | # | *-0.18* |
| gma-miR166a-3p | **-1.36** | **-0.86** | *0.58* | **0.49** | *-0.45* |
| ath-miR5658 | **-2.68** | **-1.35** | *0.29* | *0.3* | *0.04* |
| **PC-3p-7389** | **-0.97** | **-1.26** | *0.56* | *0.29* | *0.06* |
| ath-miR832-3p | **1.34** | **-1.78** | *0.37* | **0.87** | *0.1* |
| aly-miR172b | ***2.95*** | *6.03* | # | # | # |
| aly-miR319c | **0.5** | *-0.35* | **-0.78** | **-0.72** | *0.13* |
| aly-miR396b | *0.59* | *0.55* | ***-1.90*** | *-0.29* | *0.26* |
| ptc-miR172i | ***1.36*** | *0.39* | ***-0.25*** | *-0.24* | *-0.76* |
| osa-MIR1851-p5 | *-0.66* | ***-1.5*** | ***0.26*** | *0.52* | *-0.27* |
| ptc-miR156k | *-0.22* | **0.70** | **-0.88** | *-0.22* | # |
| ghr-miR156c | *-0.29* | **0.55** | **-1.01** | ***-0.86*** | *0.06* |
| ptc-miR399i | *1.04* | # | *0.72* | *0.19* | *0.23* |
| tcc-miR399g | *1.24* | # | *0.82* | *0.47* | *0.3* |
| aly-miR157a | **2.45** | *0.67* | *-0.31* | *-0.36* | *0.13* |
| ghr-miR827a | *3.03* | # | *1.36* | # | # |
| aly-miR156h | **1.16** | *0.43* | *-0.3* | *-0.08* | *-0.08* |
| ath-miR2938 | *4.95* | # | # | # | # |
| ath-miR855 | *-0.16* | **-1.25** | *1.35* | *0.48* | *-0.34* |
| csi-MIR396c-p5 | *-0.32* | **-2.05** | *0.41* | **0.34** | *-0.27* |
| aly-MIR408-p5_L | **-1.94** | **-1.69** | **0.79** | *0.53* | *-0.06* |
| **PC-3p-38876** | *-0.42* | **-2.01** | *0.06* | *0.41* | *-0.17* |
| **PC-5p-237595** | *-0.81* | ***-2.03*** | *-0.11* | *0.50* | *-0.22* |
| aly-MIR408-p5_2ss | *-0.37* | **-2.12** | *0.36* | **0.43** | *-0.26* |
| aly-MIR408-p3_2ss | *-0.71* | **-1.84** | *0.18* | *0.53* | *-0.07* |
| mtr-MIR2608-p3 | *-0.37* | **-2.15** | *0.31* | **0.48** | *-0.21* |
| sbi-MIR396c-p3 | *-0.46* | ***-1.95*** | *0.26* | **0.56** | *-0.16* |
| **csa-miRn7-5p** | *-0.43* | ***-1.84*** | -0.74 | **1.38** | ***0.85*** |
| aly-miR159a | *0.5* | *-0.02* | *-0.48* | **-0.6** | *0.08* |
| aly-miR159a stdev | *1.68* | ***6.53*** | *2.82* | # | *0.77* |
| csi-MIR3951-p3 | *-0.9* | ***-1.56*** | *-0.48* | *0.39* | # |
| bna-miR156a | *-0.27* | *0.39* | **-1.02** | ***-0.97*** | *0.4* |
| peu-MIR2911-p5 | **4.26** | **-1.86** | **1.59** | *-0.18* | **0.56** |
| ath-miR156i | **-1.65** | **-1.66** | *0.42* | *0.4* | *-0.06* |
| ath-miR2936 | **-2.12** | ***-1.07*** | *-0.12* | *0.88* | *0.50* |
| aly-miR156e | ***-0.83*** | ***-1.70*** | *0.14* | *0.57* | *0.01* |
| osa-MIR408-p3 | **-0.42** | **-1.98** | *0.31* | **0.41** | *-0.25* |
| tcc-MIR156b-p3 | **-0.51** | **-1.99** | *0.27* | *0.47* | *-0.20* |
| ath-miR3440b-3p | *0.82* | *-0.57* | # | # | *0.15* |
| aly-miR157d | **2.42** | **0.73** | *-0.29* | *-0.27* | *0.03* |
| tcc-miR156a | *-0.11* | *-0.36* | *-0.59* | *0.26* | *0.04* |
| aly-miR827 | ***3.74*** | ***1.18*** | ***2.74*** | # | # |
| ath-miR827 | *5.15* | # | *2.23* | # | # |
| aly-miR396astev | **1.46** | **2.49** | **1.3** | *-0.58* | *0.22* |
| tcc-miR396d | ***-2.02*** | *0.2* | ***-1.92*** | # | # |
| aly-miR858 | *-0.64* | ***-0.72*** | ***1.13*** | *0.66* | *-0.19* |
| ath-miR156j | **-1.93** | **-1.61** | **0.73** | *0.53* | *-0.16* |
| tcc-miR398a | ***-2.26*** | ***-1.34*** | ***-3.21*** | *-0.04* | *-0.12* |
| aly-miR167b | ***1.75*** | **5.2** | ***3.26*** | # | *0.36* |
| aly-miR319a | **0.37** | **-0.55** | **-0.89** | **-0.61** | *0.17* |
| aly-miR396a | **1.82** | *-0.15* | **-1.4** | ***-0.64*** | *-0.54* |
| aly-miR156d | ***0.95*** | ***1.76*** | ***-1.29*** | # | # |

*the value of Log_2_ (hybridization signal from inoculated sample / hybridization signal from non-inoculated sample).

Boldface, p-value < 0.01, signal > 500. Boldface and superior, p-value < 0.01, signal < 500. Superior, p-value > 0.01. '#', no hybridization signal detected.

PC, predicted candidate.

dpi, days post inoculation for leaf samples.

mfi, male flowers.

ffi, female flowers.
