# Supplementary material for: High-Throughput Sequencing Identifies Novel and Conserved Cucumber (Cucumis sativus L.) microRNAs in Response to Cucumber Green Mottle Mosaic Virus Infection
Source: PLoS One. 2015 Jun 15;10(6):e0129002. doi: 10.1371/journal.pone.0129002 (PMC4468104; doi:10.1371/journal.pone.0129002)
Supplement: S4 Table — (DOC) [file pone.0129002.s006.doc]

**Table S4.** GO analysis indicating the biochemical roles (p <0.01) for the target genes of the 88 miRNAs expressed during the hybridization analysis of CGMMV-infected cucumbers using miRNA sequences from closely related plant species.

| Sample | GO ID | Gene count | GO name | GO function | P-value |
| --- | --- | --- | --- | --- | --- |
| dpi* | 0035299 | 1 | Inositol pentaphosphate 2-kinase activity | Molecular function | 0.003903 |
|  | 0019901 | 1 | Protein kinase binding | Molecular function | 0.003903 |
|  | 0000462 | 1 | Maturation of SSU-rRNA from tricistronic rRNA transcript | Biological process | 0.003903 |
|  | 0001906 | 1 | Cell killing | Biological process | 0.003903 |
|  | 0004000 | 1 | Adenosine deaminase activity | Molecular function | 0.003903 |
| 10 dpi | 0005834 | 13 | Heterotrimeric G-protein complex | Cellular component | 0.000183 |
|  | 0008375 | 7 | Acetylglucosaminyl transferase activity | Molecular function | 0.000387 |
|  | 0050661 | 4 | NADP binding | Molecular function | 0.003965 |
|  | 0004616 | 4 | Phosphogluconate dehydrogenase (decarboxylating) activity | Molecular function | 0.003965 |
|  | 0006487 | 5 | Protein N-linked glycosylation | Biological process | 0.004712 |
|  | 0007169 | 9 | Transmembrane receptor protein tyrosine kinase signaling pathway | Biological process | 0.007583 |
| 30 dpi | 0003674 | 778 | Molecular function | Molecular function | 1.13E-05 |
|  | 0005575 | 541 | Cellular component | Cellular component | 2.50E-05 |
| 50 dpi | 0005834 | 13 | Heterotrimeric G-protein complex | Cellular component | 0.0067581 |
|  | 0006468 | 47 | Protein phosphorylation | Biological process | 0.0098345 |
| mfi and ffi vs. mfn and ffn | 0045454 | 1 | Cell redox homeostasis | Biological process | 0.004125 |
|  | 0005840 | 46 | Ribosome | Cellular component | 0.005435 |
|  | 0005774 | 36 | Vacuolar membrane | Cellular component | 0.008933 |
| ffi | 0004402 | 2 | Histone acetyltransferase activity | Molecular function | 7.12E-05 |
|  | 0008152 | 4 | Metabolic process | Biological process | 0.001661 |
|  | 0005777 | 2 | Peroxisome | Cellular component | 0.001931 |
|  | 0008168 | 2 | Methyltransferase activity | Molecular function | 0.007937 |
|  | 0006099 | 1 | Tricarboxylic acid cycle | Biological process | 0.008642 |
|  | 0000234 | 1 | Phosphoethanolamine N-methyltransferase activity | Molecular function | 0.008642 |
|  | 0030246 | 1 | Carbohydrate biosynthetic process | Molecular function | 0.008642 |
| dpi* vs. mfi and ffi | 0003677 | 77 | DNA binding | Molecular function | 0.003819 |
| ffi vs. mfi | 0003674 | 177 | Molecular function | Molecular function | 7.47E-06 |
|  | 0008150 | 191 | Biological process | Biological process | 0.001131 |
|  | 0008270 | 41 | Zinc ion binding | Molecular function | 0.003241 |
|  | 0005575 | 118 | Cellular component | Cellular component | 0.00385 |
|  | 0048235 | 3 | Pollen sperm cell differentiation | Biological process | 0.004635 |
|  | 0000287 | 6 | Magnesium ion binding | Molecular function | 0.007866 |

dpi, days post inoculation for leaf samples. dpi*, the leaf samples from 10, 30 and 50 days post inoculation with CGMMV were pooled and used to probe the hybridization chip. mfi, male flowers from inoculated cucumber plants. ffi, female flowers from inoculated cucumber plants. mfn, male flowers from non-inoculated cucumber plants. ffn, female flowers from non-inoculated cucumber plants. vs., compared to.
