# Supplementary material for: High-Throughput Sequencing Identifies Novel and Conserved Cucumber (Cucumis sativus L.) microRNAs in Response to Cucumber Green Mottle Mosaic Virus Infection
Source: PLoS One. 2015 Jun 15;10(6):e0129002. doi: 10.1371/journal.pone.0129002 (PMC4468104; doi:10.1371/journal.pone.0129002)
Supplement: S5 Table — (DOC) [file pone.0129002.s007.doc]

**Table S5.** KEGG analysis identifying biochemical pathways (p <0.1) for the target genes of 88 miRNAs expressed during the hybridization analysis of CGMMV infected cucumbers using miRNA sequences from closely related plant species.

| Sample | Pathway ID | Gene count | KEGG description | P-value |
| --- | --- | --- | --- | --- |
| 10 dpi | 480 | 5 | Glutathione metabolism | 0.017321 |
|  | 564 | 3 | Glycerophospholipid metabolism | 0.019353 |
|  | 30 | 5 | Pentose phosphate pathway | 0.036285 |
|  | 1100 | 42 | Metabolic pathways | 0.052644 |
|  | 510 | 5 | N-Glycan biosynthesis | 0.064255 |
|  | 40 | 4 | Pentose and glucuronate interconversions | 0.088987 |
| 30 dpi | 3015 | 12 | mRNA surveillance pathway | 0.068416 |
| 50 dpi | 10 | 12 | Glycolysis / Gluconeogenesis | 0.017348 |
|  | 230 | 11 | Purine metabolism | 0.019158 |
|  | 564 | 3 | Glycerophospholipid metabolism | 0.042715 |
|  | 620 | 8 | Pyruvate metabolism | 0.044186 |
|  | 510 | 6 | N-Glycan biosynthesis | 0.052318 |
|  | 4120 | 4 | Ubiquitin mediated proteolysis | 0.053537 |
| ffi | 310 | 1 | Lysine degradation | 0.041091 |
|  | 20 | 1 | Citrate cycle (TCA cycle) | 0.054483 |
| dpi*vs. mfi and ffi | 510 | 8 | N-Glycan biosynthesis | 0.014739 |
|  | 3008 | 17 | Ribosome biogenesis in eukaryotes | 0.086720 |
| ffi vs. mfi | 710 | 8 | Carbon fixation in photosynthetic organisms | 0.00026 |
|  | 10 | 8 | Glycolysis / Gluconeogenesis | 0.001117 |
|  | 620 | 6 | Pyruvate metabolism | 0.002968 |
|  | 230 | 7 | Purine metabolism | 0.004169 |
|  | 1100 | 21 | Metabolic pathways | 0.042985 |
|  | 1110 | 12 | Biosynthesis of secondary metabolites | 0.050198 |
|  | 3008 | 7 | Ribosome biogenesis in eukaryotes | 0.054816 |
|  | 20 | 2 | Citrate cycle (TCA cycle) | 0.083697 |

dpi, days post inoculation for leaf samples. mfi, male flowers from inoculated cucumber plants. ffi, female flowers from inoculated cucumber plants. dpi*, the leaf samples of 10, 30 and 50 days post inoculation with CGMMV were pooled and used to probe the hybridization chip. vs., compared to.
